# Lim Homeobox 8 Is Essential for Beta Adrenergic Stimulation of Thermogenesis in Human Adipocytes

**DOI:** 10.3390/cells15111000

**Published:** 2026-05-29

**Authors:** Katalin Gyurina, Ádám Radványi, László Sasi-Szabó, Enikő Felszeghy, Emese Rácz, Gábor Méhes, Andrea Kádár, Csaba Fekete, Tamás Röszer

**Affiliations:** 1 Department of Pediatrics, Faculty of Medicine, University of Debrecen, 4032 Debrecen, Hungaryfelszeghy.eniko@med.unideb.hu (E.F.); 2 Kálmán Laki Doctoral School, University of Debrecen, 4032 Debrecen, Hungary; 3 Department of Pathology, Faculty of Medicine, University of Debrecen, 4032 Debrecen, Hungary; 4 Laboratory of Integrative Neuroendocrinology, Hun-Ren Institute of Experimental Medicine, 1083 Budapest, Hungary

**Keywords:** obesity, adipocyte, thermogenesis, uncoupling protein 1, interleukin-33

## Abstract

**Highlights:**

- LHX8 is essential for catecholamine-induced thermogenesis
- Lack of LHX8 may contribute to impaired thermogenic responsiveness in obesity
- The LHX8–IL-33 pathway may represent a potential therapeutic target to overcome catecholamine resistance and support obesity treatment

**Abstract:**

Catecholamines are crucial signaling molecules that initiate thermogenesis in adipocytes through beta-adrenergic receptors (ADRBs). Adipocyte catecholamine resistance is a common feature of pediatric obesity, often impeding weight loss and the maintenance of a healthy body fat percentage. Our aim was to identify possible mechanisms that may be responsible for the development of catecholamine resistance in adipocytes. We demonstrate that Lim homeobox 8 (LHX8), a transcription factor previously known for its role in gametogenesis, is essential for catecholamine-induced thermogenesis in human adipocytes. LHX8 is expressed in developing human adipocytes throughout intrauterine and perinatal life, as well as in adulthood, and its expression levels positively correlate with the expression of key thermogenesis genes. Pediatric obesity diminished adipocyte expression of LHX8. Functionally, ADRB stimulation failed to induce thermogenesis in both mouse and human adipocytes when LHX8 was absent. Conversely, LHX8 overexpression enhanced thermogenesis in murine adipocytes. Mechanistically, LHX8 stimulated adipocyte interleukin-33 (IL-33) synthesis in response to ADRB activation, which subsequently increased thermogenic gene expression in both human and mouse adipocytes. In conclusion, adipocyte LHX8 is indispensable for catecholamine-responsive thermogenesis and represents a promising novel therapeutic target to overcome catecholamine resistance and promote effective weight management.

## 1. Introduction

Obesity is the most common non-communicable disease globally [1]. The prevalence of obesity is rising rapidly in children, affecting almost 20% of children globally, and leading to projections that >57% of children will be obese as adults in the coming decade [1]. Developing obesity at an early age accelerates the progression of obesity-associated diseases such as insulin resistance, type 2 diabetes, metabolic syndrome, and cardiovascular and renal diseases. Maintaining or restoring healthy body adiposity early in life is crucial for preventing the detrimental long-term health consequences linked to pediatric obesity [2]. However, pharmacological support of weight management is limited in childhood, making it timely and relevant to identify molecular targets of future therapy of pediatric obesity.

Obesity arises from the excessive expansion of adipose tissue, a condition where lipid storage dominates over lipid catabolism within adipocytes. A decrease or absence of adipocyte thermogenesis accelerates this lipid storage [3]. Conversely, promoting adipocyte thermogenesis may facilitate the breakdown of excess stored fat [3]. Adipocyte thermogenesis is the result of mitochondrial uncoupling, which dissipates chemical energy as heat rather than ATP. This process is further supported by an expansion of the adipocyte mitochondrial network, which increases fatty acid oxidation [3].

Adipocyte thermogenesis is robust in the subcutaneous adipose tissue (SAT) during the perinatal period, when lipid-fueled heat production is vital for maintaining the core body temperature of newborns [4]. These thermogenic adipocytes—known as brown, beige or brite (brown-in-white) adipocytes—persist in SAT throughout childhood, and their premature loss is associated with pediatric obesity [5,6]. Because restoring the thermogenic competence of subcutaneous adipocytes could help mitigate childhood obesity, significant research efforts are focused on understanding their development and identifying molecules for potential clinical application to stimulate their differentiation and function [3].

Catecholamines are important signaling molecules that activate lipolysis and thermogenesis in adipocytes via beta-adrenergic receptors (ADRBs) [6]. Adipocyte resistance to catecholamines is common in obesity and may impede successful weight loss [7,8]. Given the low success rate for pediatric patients with obesity in achieving and maintaining a healthy body fat percentage [1], understanding the mechanisms that impede catecholamine sensitivity is critical.

Lim homeobox 8 (LHX8), a transcription factor, is expressed in murine brown adipocytes and in human thermogenic (beige or brite) adipocytes and is associated with the expression of LIM domain-binding protein 1, a key regulator of brown adipocyte functioning [9,10,11,12]. Although initially identified for its roles in oocyte maturation and neuronal development, its function in adipocytes remains unclear. Crucially, LHX8 expression is absent in both human [13] and mouse [4] white adipose tissue (WAT), also known as depot fat. We previously demonstrated that LHX8 expression levels are positively correlated with the expression of genes involved in thermogenesis and mitochondrial biogenesis within human adipose tissue [4]. Conversely, conditions such as obesity are associated with reduced LHX8 expression; for example, in adipocytes differentiated in vitro from adipogenic precursors of obese individuals [14], and a similar loss of adipocyte LHX8 expression is observed in pediatric obesity [5]. In this study, we explored whether LHX8 is involved in adipocyte functioning and its deficiency may play a role in the development of pediatric obesity.

## 2. Materials and Methods

### 2.1. Human Samples

Subcutaneous adipose tissue (SAT) specimens from the abdominal-inguinal regions (Supplemental Figure S1A) were obtained from newborns, infants, children, and adolescents undergoing elective surgeries at the Department of Pediatrics, University of Debrecen (Supplemental Table S1). Venous blood samples were drawn into EDTA-coated hematological tubes during these procedures. Clinical data, including disease history and anthropometric measurements (body weight, height, skin fold thickness, and, when possible, abdominal fat thickness), were also recorded. Abdominal fat thickness was measured via abdominal ultrasonography performed pre-operatively (Supplemental Figure S1B). Ultrasound examinations were performed using a Philips ultrasound machine by a trained pediatric radiologist. The transducer was applied gently during the examination to ensure that the saved images were suitable for accurate measurement of abdominal fat thickness. Abdominal fat tissue thickness was measured at three different points. and the average of these measurements represented the patient’s abdominal fat thickness.

Anthropometric data were analyzed using the World Health Organization (WHO) AnthroPlus software (WHO, Geneva, Switzerland, Windows Desktop Version 10.0.3), which provided standardized indicators for obesity status based on WHO growth reference data. We determined age- and sex-specific percentiles and *z*-scores for HFA, WFA, and BMI-for-age. For morbidly obese cases who exceeded the anthropometric limits of the WHO pediatric reference population, the Centers for Disease Control and Prevention (CDC) growth charts (CDC, Atlanta, GA, USA) were employed as a complementary reference. The combined use of these references ensured a consistent and comprehensive evaluation of anthropometric status across the entire study population.

Adipose tissue samples were processed for RNA isolation, histological analysis, and adipocyte isolation [4]. Isolated adipocytes were subsequently analyzed by flow cytometry (FACS) or cultured in vitro, as described [4]. Plasma samples were separated from whole blood via centrifugation and used for ELISA analyses. Prior to sample collection, written informed consent was obtained from the parents or guardians of all patients included in the study. The study was conducted in strict accordance with the ethical guidelines of the Declaration of Helsinki. Participants were excluded from the study if they presented with any of the following conditions: severe chronic or inflammatory diseases, acute or chronic infections, oncological conditions, or clotting disorders. Further exclusion criteria included any complications appearing during the surgical procedure, ongoing drug treatment at the time of the study, and withdrawal of consent by the parents or guardians. All patients were confirmed to be negative for severe acute respiratory syndrome coronavirus 1 (SARS-CoV-1), human immunodeficiency virus, and hepatitis A and B.

Adipose tissue specimens were also procured post-mortem (during autopsies) from fetuses, infants, children and adults at the Department of Pathology, University of Debrecen (Supplemental Table S2), and utilized for histology analyses.

Sex was considered as a biological variable in this study, with patients recruited from both sexes. However, male patients were overrepresented due to several factors, including sex ratio differences at birth [15], sex-dependent differences in the prevalence of indications for surgical interventions [16], sex-specific differences in the indications for second-trimester abortions, and sex-specific mortality rates during the third trimester [17]. This study population was different from the populations we analyzed in our previous studies on LHX8 expression [4,18].

### 2.2. Animals and In Vitro Assays

We used wild-type C57BL/6 male and female mice (Charles River Laboratories, Wilmington, MA, USA), housed under specific pathogen-free (SPF) conditions. Mice were housed individually under standard environmental conditions (12 h dark/light cycle, lights on at 6 a.m., temperature 22 ± 1 °C, humidity 40–60%) and were provided standard chow diet (S8189-S095, SSNIFF Spezialdiäten, Soest, Germany) and water ad libitum. *Lhx8* deficiency was locally induced by injecting the left inguinal adipose tissue depot (iAT) with 1 μg/kg body weight *Lhx8* siRNA (Ambion Silencer Pre-designed *Lhx8* siRNA, Thermo Fisher Scientific, Waltham, MA, USA), using magnetofection with Turbofectamine transfection reagent, as described [4]. As a control, we used a non-silencing siRNA (Thermo Fisher Scientific) injected into the right iAT. Briefly, siRNA delivery into the adipocyte cytosol was achieved via magnetofection, using siRNA–magnetic nanoparticle complexes (In Vivo DogtorMag, OzBiosciences, San Diego, CA, USA). The *Lhx8* siRNA complexes were injected into the right iAT, while the non-silencing siRNA into the left iAT, and delivery of the magnetic nanoparticles was facilitated by magnetic exposure of the iAT [19].

For LHX8 overexpression, mice were transfected by injecting their right iAT with an LHX8 DNA plasmid (LHX8 Human Tagged ORF Clone, mGFP tagged, pCMV6-Entry, mammalian vector, OriGene Technologies, Inc., Rockville, MD, USA) via magnetofection with Turbofectamine, as described [4]. An empty pCMV6 transfection vector (OriGene Technologies, Inc., InvivoGene, Toulouse, France) was used as a control and injected into the left iAT. To assess the effect of systemic IL-33 administration, mice were injected subcutaneously with 0.4 μg recombinant human IL-33 daily for 4 days (ImmunoTools GmbH, Friesoythe, Germany), as described [20].

Primary mouse preadipocytes were isolated from iAT depots via collagenase digestion and subsequent separation of cell fractions, and then analyzed or cultured, as described [4]. Primary mouse preadipocytes and 3T3-L1 preadipocytes were cultured in high-glucose Dulbecco’s modified Eagle medium (DMEM) supplemented with 10% fetal bovine serum, 1% L-glutamine and 20 µg/mL insulin (I9278, Merck, Rahway, NJ, USA). Cells were differentiated into adipocytes, as described [4], and treated with 100 μM isoproterenol (Cat. No. 15627, Sigma-Aldrich, Darmstadt, Germany), 50 μM forskolin (Cat. No. 344282, Merck, Darmstadt, Germany), 500 μM 8-bromo-cAMP (Br-cAMP, Cat. No. B5386, Merck), 30 ng/mL recombinant IL-33 (Cat. No.12340332, ImmunoTools GmbH, Friesoythe, Germany), 10 μM JSH-23 (Cat. No. 749886-87-1, Cayman Chemicals) or 200 nM CREB inhibitor 666-15 (Cat. No. HY-101120, MedChemExpress) and 100 nM STAT6 phosphorylation inhibitor AS1517499 (Cat. No. 7636, Tocris Bioscience, Minneapolis, MN, USA) for 4–48 h. Primary human adipocytes were isolated from the SAT biopsies using the same protocol and treatment. To inhibit LHX8 expression in human adipocytes, we transfected human SAT-derived adipocytes with a transfection reagent or *LHX8* siRNA (Thermo Fisher Scientific) in vitro.

We also studied two murine models of obesity. Adult C57Black/6 mice at age 8 weeks were rendered obese with high-fat diet (HFD) feeding for 12 weeks, using a rodent HFD (SSNIFF Spezialdiäten). Leptin-resistant diabetic obese (*Lepr^db^*^/*db*^) mice (Janvier Labs, Le Genest-Saint-Isle, France) were used to isolate iAT specimens at age 12 weeks.

### 2.3. RNA Isolation and qPCR

Extraction of total RNA from tissues or cells was performed using TRIzol reagent (Merck Sigma-Aldrich, St. Louis, MO, USA), as described [4]. RNA quantity was measured using the NanoDrop 2000/2000c spectrophotometer (Cat. No. 1.6.198, Thermo Fisher Scientific). For cDNA synthesis, we used a high-capacity cDNA reverse transcription kit (Cat. No. 4368813, Thermo Fisher Scientific). Quantitative PCR assays were carried out on an Analytik Jena platform (Jena, Germany), with 45 cycles, using qPCRsoft 4.1 Analytic Jena software for analysis. Forward and reverse primers were custom-synthesized (Merck). Primer sequences are summarized in Supplemental Tables S3 and S4. We used my-Budget EvaGreen^®^ QPCR-Mix II with ROX (BioBudget, Krefeld, Germany) master mix for the qPCR assays. The mean threshold cycle (CT) value for *ACTB* and *GAPDH* was used for human samples, and *Actb* and *Gapdh* for murine samples, as references. We calculated relative gene expression values using the ΔCT method [21] and were displayed in logarithmic format. Changes in gene expression levels were assessed by the ΔΔCT method and were expressed as fold change [21].

### 2.4. Next-Generation RNA Sequencing

A BGISEQ-500 platform was used for next-generation RNA sequencing (BGI Genomic Services, Beijing, China), yielding an average of ~26.20 M reads per sample. We used SAT samples from human male infants and children (n = 3/3); 3/3 samples from wild-type and *Lhx8* siRNA-transfected, in vitro cultured, mouse adipocytes, and 3/3 iAT depots from C57BL/6 mice at postnatal days 6 and 56. We used HISAT (hierarchical indexing for spliced alignment of transcripts) and Bowtie2 to align clean reads to the reference genome [22,23], and RSE was used to determine gene expression levels. We used EnrichR and Interferome 2.0 to annotate transcripts [24,25]. For visualization of gene expression changes, we generated clustered image maps using CIMminer [26,27]. Predicted protein–protein interaction networks were rendered with the STRING Functional Protein Associations Network [28].

### 2.5. Histology, Immunohistochemistry, and Image Analysis

Tissue specimens were fixed in 4% paraformaldehyde, dehydrated, embedded in paraffin, and 5 μm thin sections were made. We stained sections with hematoxylin and eosin (H&E) or Masson’s trichrome (BioGnost, Zagreb, Croatia). Immunohistochemistry was performed with antibodies listed in Supplementary Table S5. For antigen retrieval, we used a citrate-based antigen unmasking solution (Vector Laboratories, Newark, CA, USA), incubating sections at 95 °C for 10 min to facilitate epitope exposure. Following incubation, slides were allowed to cool gradually to room temperature in the retrieval buffer before proceeding with the subsequent staining protocol. We used a horseradish peroxidase-conjugated secondary antibody and diaminobenzidine to visualize primary antibody binding (HISTOLS^®^- DAB, Histopathology Ltd., Pécs, Hungary). Adipocyte contours were visualized by H&E staining and perilipin-1 (PLIN1) immunohistochemistry. Histomorphometry analysis was performed using Fiji (ImageJ distribution, version 1.54p). Image processing steps were standardized: scale calibration was performed, images were then converted to 8-bit grayscale to normalize intensity values, a threshold was applied to enhance contrast and facilitate the identification of structures of interest, and individual cells were then manually outlined using the freehand selection tool. Slides were scanned with a Panoramic 480 Digital Slide Scanner (3DHistotech Ltd., Budapest, Hungary).

For each selected adipocyte, cell area and perimeter were measured. For transmission electron microscopy (TEM), tissue specimens were fixed in paraformaldehyde/glutaraldehyde. For fluorescent microscopy of mitochondrial content and morphology, we cultured preadipocytes on glass coverslips or glass-bottom plates (Greiner Bio-One GmbH, Frickenhausen, Germany). MitoTracker Red (Thermo Fisher Scientific) was used to stain mitochondria.

### 2.6. Fluorescence-Activated Cell Sorting (FACS)

SAT samples were subjected to collagenase digestion, and adipocytes and stromal vascular fraction cells were isolated, as described [4]. Adipocytes were fixed with 0.5% paraformaldehyde for 1 h, treated with cell eBioscience permeabilization buffer (Thermo Fisher Scientific) and labeled with antibodies raised against mouse and human CD36, LHX8, IL-33, ADRB2, PLIN1, IL-13, IL-4, IFNβ, STAT6, phosphorylated STAT6, c-JUN, phosphorylated c-JUN, and p38 MAPK (Supplemental Table S5), and analyzed with a BD LSR II flow cytometer to determine mean fluorescence intensity (MFI) values in specific cell populations (BD Biosciences, Franklin Lakes, NJ, USA). FACS Diva v8.0 (BD Biosciences) and FlowJo10.8.1. (FlowJo LLC, Ashland, OR, USA) were used for analysis. Relative cell size was measured using forward scatter area (FSC-A). Mitochondrial content was analyzed with MitoTracker Red (Thermo Fisher Scientific), as described [4]. Lipid droplets were stained with HCS LipidTOX™ red neutral lipid stain (Cat. No. H34476, Thermo Fisher Scientific). We used 0.1 ng/mL MitoThermo Yellow (MTY), a temperature-sensitive fluorescent probe [29] to label 10^6^/mL cells and assess mitochondrial thermogenesis and uncoupling, as described [30]. MTY was developed and provided by Dr. Y-T. Chang (Pohang University of Science and Technology, Republic of Korea).

### 2.7. ELISA and Photometric Assays

The IL-33 levels of human and mouse plasma were measured with commercial ELISA kits (Cat. No. RB0815, Merck). Mitochondrial respiration was evaluated with the colorimetric WST-81 assay (Carl Roth, Karlsruhe, Germany), measuring NADH-DH activity, as described [31].

### 2.8. Indirect Calorimetry

One week before the experiment, 60-day-old C57/BL6 male mice (n = 16, 8/group) were individually housed in training boxes for adapting to social isolation and the design of the boxes. We used training boxes that were designed in the same way as the measurement boxes, with the only difference that the boxes were not connected to the measuring equipment. After this acclimatization period, the mice and their bedding material were placed in the calorimetric cages of the TSE Phenomaster setup (TSE Systems, Berlin, Germany). The data of O_2_-consumption and CO_2_-production were collected every 15 min by the TSE Phenomaster apparatus. Estimated energy expenditure (EE, kcal/h) was calculated automatically by the TSE Labmaster software V8.0.3 based on the Weir equation: EE = [3.941 (VO_2_) + 1.106 (VCO_2_)] × 1.44 in every 15 min cycle. After indirect calorimetry was performed at room temperature (21 ± 1 °C), the measurement was repeated under thermoneutral conditions (30 ± 1 °C) in the climatic chamber of the TSE Phenomaster system. The full raw dataset is available in Supplementary File S1.

### 2.9. Data Representation and Statistics

Data are represented as mean ± S.E.M. Data analysis and visualization were performed using GraphPad Prism 5.0 statistical software (San Diego, CA, USA). Gene expression values were analyzed using linear regression models. Because gene expression variables showed right-skewed distributions, values were log10-transformed prior to analysis. Associations between *LHX8* mRNA expression and its target genes were first assessed using univariable linear regression. To evaluate potential confounding effects of age and sex, multivariable linear regression models were subsequently fitted, including age (months) and sex (female/male) as covariates. Confounding was assessed by comparing the regression coefficient (β) for *LHX8* mRNA level before and after adjustment for age and sex.

CIM Miner was used to generate heat maps (so-called one-matrix clustered image maps) [27] with the Euclidean distance algorithm. The cluster method was average linkage, while the binning method was equal width. We used an unpaired, 2-tailed Student’s *t*-test to determine statistical differences between groups with a normal distribution. The 2-tailed Pearson test with Gaussian *p*-value approximation was used for correlation analysis between two variables, with a 95%confidence interval (α = 0.05). The number of biological replicates, statistical tests, and *p* values are defined in the figures and figure legends. The DEseq2 algorithm was used to identify DEGs between sample groups in NGS analysis (*p* < 0.05) [26].

## 3. Results

### 3.1. Human Preadipocytes and Adipocytes Express LHX8

We studied the distribution of LHX8-expressing cells in developing human SAT across intrauterine and postnatal life, using specimens obtained from the abdominal wall (Supplemental Figure S1A–D). Thermogenic, multilocular, UCP1^+^ adipocytes were detectable in the SAT depots during the perinatal period and throughout childhood (Supplemental Figure S1E). As controls for LHX8 immunohistochemistry, we used human fetal spinal cord and skeletal muscle (Supplemental Figure S2A–C).

**Figure 1:**
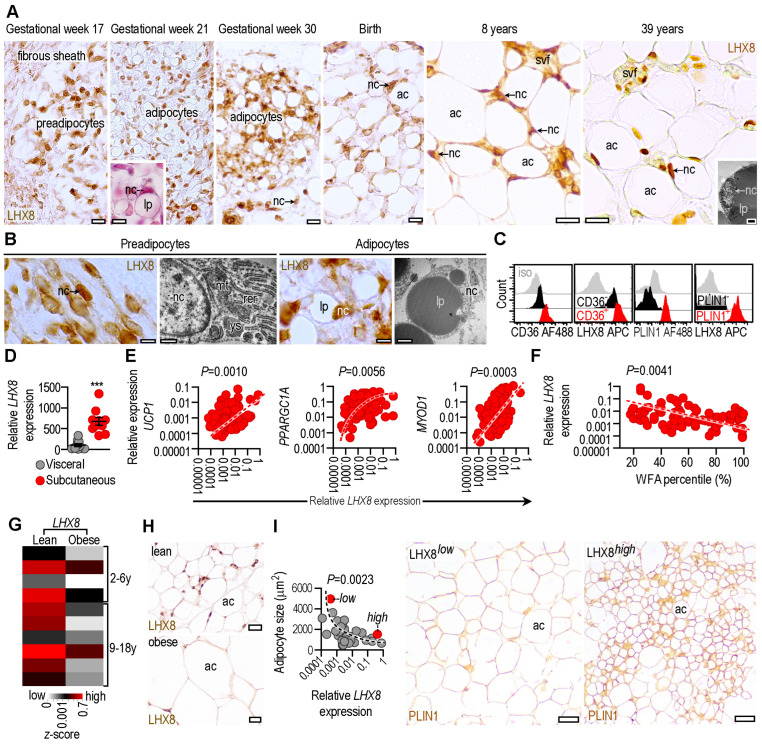
LHX8 expression in human adipose tissue. (**A**) LHX8 immunostaining of human subcutaneous adipose tissue (SAT) samples at gestational weeks 17, 21, 30; at birth and at 8 and 39 years of age. Inlet shows Masson trichrome staining. nc: nucleus, ac: adipocyte, svf: stromal vascular fraction, lp: lipid droplet, scale bar 30 μm. (**B**) LHX8 immunostaining and electron microscopy of preadipocytes and adipocytes of a human fetus. nc: nucleus, mt: mitochondria, rer: rough endoplasmic reticle, lys: lysosome, scale bars 5 μm and 500 nm. (**C**) Flow cytometry histograms of human adipocytes labeled with antibodies against LHX8, CD36, and PLIN1. Male donor at 23 months of age. (**D**) Relative *LHX8* mRNA expression of human visceral (mesoappendix) and subcutaneous adipose tissue, from donors at 3–17 years of age. (**E**) Correlation of *LHX8* and *UCP1*, *PPARGC1A* and *MYOD1* mRNA levels in SAT of children (males, aged 2 months–17 years, n = 130). Relative expression levels are shown on a logarithmic scale. (**F**) Correlation of adipose tissue *LHX8* mRNA levels and WFA percentiles (males, age 2–17 years, n = 86). (**G**) Adipose tissue *LHX8* expression in lean and obese children from two age groups (2–6 years and 9–18 years). (**H**) LHX8 immunostaining of lean and obese SAT (age 5 and 4.8 years, respectively). ac: adipocyte, scale bar 30 μm. (**I**) Correlation of adipocyte size and adipose tissue *LHX8* expression in the SAT. Each data point represents one patient. PLIN1 immunostaining of the SAT in two patients with low and high *LHX8* expression levels. Age: 3 months, scale bar 30 μm. *** *p* < 0.001, unpaired, 2-tailed, unpaired Student’s *t*-test (**D**); univariable linear regression analysis and Spearman correlation analysis with Gaussian approximation (**E**,**F**,**I**).

Preadipocytes were first identified in the second trimester and exhibited strong nuclear immunostaining for LHX8 (Figure 1A and Figure S2D,E). Similarly, adipocyte nuclei in the human fetus showed positive LHX8 expression throughout the third trimester (Figure 1A and Figure S2F–H). Intrauterine growth restriction had no discernible effect on adipocyte LHX8 expression (Supplemental Figure S2I). Furthermore, strong nuclear LHX8 immunolabeling was consistently detectable in adipocytes at birth and persisted through childhood and adulthood (Figure 1A).

Nuclear LHX8 expression was observed in both preadipocytes and mature adipocytes within human SAT (Figure 1B). LHX8 expression was associated with both the human preadipocyte/adipocyte marker cluster of differentiation 36 (CD36) [32] and the adipocyte marker perilipin (PLIN1) [33] (Figure 1C), with slightly higher *LHX8* mRNA levels in mature adipocytes (Supplemental Figure S3A).

*LHX8* mRNA expression was stronger in SAT than in visceral fat (Figure 1D). Comparative analysis showed that human adipocytes, murine adipocytes, and the 3T3-L1 mouse preadipocyte cell line expressed similar levels of LHX8 protein (Supplemental Figure S3B), allowing the use of primary human and mouse adipocytes, as well as 3T3-L1 cells.

### 3.2. LHX8 Is Associated with Thermogenic Gene Expression

To investigate the function of LHX8 in adipocytes, we first measured *LHX8* mRNA levels in abdominal SAT samples of children, aged 2 months–17 years (N = 149, males N = 130, females N = 19). The over-representation of male participants is due to sex-specific differences in the necessity of surgical interventions; specifically, orchidopexy and a 3–6-fold higher susceptibility to inguinal and umbilical herniation in males at an early age of 13. Despite this gender imbalance, the range of adipose tissue *LHX8* mRNA expression was similar in both groups (Supplemental Figure S3C). *LHX8* mRNA expression showed a significant positive association with the expression of three key genes after adjustment for age and sex: *UCP1* (encoding uncoupling protein 1), a central gene product for mitochondrial uncoupling; *PPARGC1A* (encoding peroxisome proliferator-activated receptor gamma coactivator 1-alpha), which is necessary for mitochondrial biogenesis; and *MYOD1* (encoding myogenic differentiation 1), which is associated with thermogenic adipocyte differentiation in the adipose tissue, along with myogenic cell fate [34] (Figure 1E). *LHX8* expression did not correlate with the expression of the key lipolysis genes *ATGL* (adipose triglyceride lipase) and *MGLL* (monoacylglycerol lipase) (Supplemental Figure S3D).

### 3.3. Pediatric Obesity Diminishes Adipose Tissue LHX8 Expression

The expression of *LHX8* was negatively associated with the weight-for-age (WFA) percentile (Figure 1F), a recognized predictor of pediatric obesity [35] (Supplemental Figure S3E). Other anthropometric values, including age and height-for-age (HFA), showed no correlation with adipose tissue *LHX8* mRNA expression (Supplemental Figure S3F).

When we compared age- and gender-matched SAT samples from children living with overweight and obesity (falling in the 95th or higher WFA percentile) and their lean peers (falling in the 25th–70th WFA percentile), we found that LHX8 mRNA levels were markedly diminished in obesity (Figure 1G). Indeed, LHX8 protein expression was absent in severely obese SAT (Figure 1H), and adipocyte size negatively correlated with adipose tissue *LHX8* mRNA levels (Figure 1I). The lack of LHX8 immunostaining in obesity was a consistent finding, observed not only in the SAT of obese patients (Figure 1H), but also in the SAT from obese, leptin-resistant (*Lepr^db^*^/*db*^) mice, and mice rendered obese by a high-fat diet (Supplemental Figure S3G).

In summary, the absence of LHX8 expression in the SAT of children is associated with adipocyte hypertrophy and a concurrent loss of thermogenic gene expression.

### 3.4. Diminished LHX8 Expression Leads to Adipose Tissue Expansion

**Figure 2:**
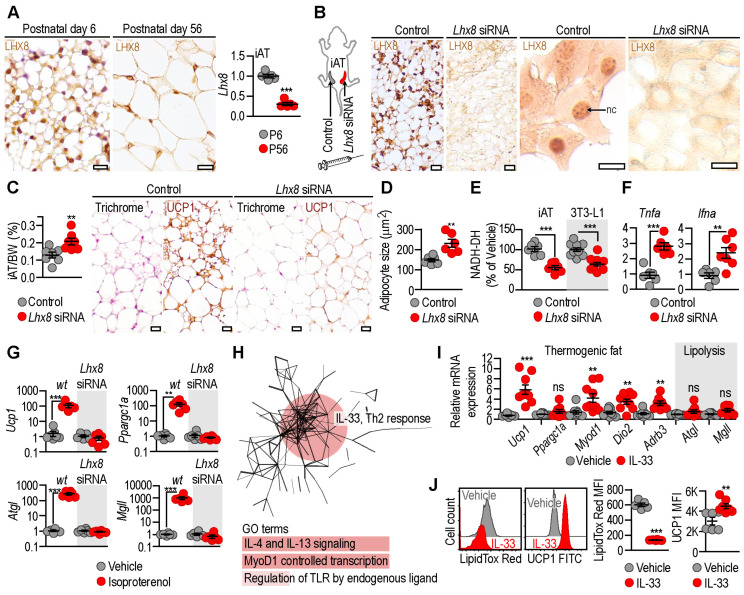
Effect of LHX8 deficiency on the adipose tissue in mouse. (**A**) LHX8 immunostaining and *Lhx8* mRNA expression levels in the inguinal adipose tissue (iAT) depot of mice at postnatal day 6 (P6) and postnatal day 56 (P56). Scale bar 30 μm. (**B**) The right iAT depot was transfected with non-silencing siRNA (control), while the left iAT with *Lhx8* siRNA on P6 (the scheme shows ventral view). LHX8 immunostaining of the iAT depots and isolated adipocytes on P10. nc: nucleus, scale bar 30 μm and 10 μm. (**C**) Ratio of iAT weight and body weight (BW) at P10. Masson’s trichrome staining and UCP1 immunostaining of iAT at P10, scale bar 30 μm. (**D**) Adipocyte size at P10. (**E**) NADH dehydrogenase activity (NADH-DH) of isolated iAT adipocytes at P10. As a comparison, 3T3-L1 cells were transfected with non-silencing siRNA (control) and *Lhx8* siRNA for three days and NADH-DH activity was then measured. (**F**) Expression of the inflammatory cytokines tumor necrosis factor alpha (*Tnfa*) and interferon alpha (*Ifna*) in mouse primary adipocytes transfected with non-silencing siRNA (control) or *Lhx8* siRNA. (**G**) Mouse primary adipocytes were treated with isoproterenol for 48 h and the mRNA expression of *Ucp1*, *Ppargc1a*, *Atgl*, and *Mgll* was measured. We used non-silencing siRNA-transfected (*wt*) and *Lhx8* siRNA-transfected cells in this assay. (**H**) Protein–protein interaction map and gene ontology (GO) terms of gene products repressed by *Lhx8* siRNA transfection in mouse adipocytes. Raw data are available at NIH GEO under accession number GSE329222. (**I**) Effect of IL-33 treatment on the expression of thermogenic and lipolytic genes in mouse adipocytes. (**J**) Flow cytometry analysis of the lipid content (measured by LipidTox Red mean fluorescence intensity (MFI)) and UCP1 protein level in mouse adipocytes treated with vehicle or IL-33. ** *p* < 0.01, *** *p* < 0.001 Student’s 2-tailed, unpaired *t*-test (**C**–**F**,**I**,**J**) or one-way ANOVA with Dunnett’s post hoc test (**G**).

We continued to define the effect of diminished LHX8 protein expression on adipose tissue development using a mouse model. Subcutaneous thermogenic adipocytes appear in the early postnatal life of mice [36]. Consistent with this developmental stage, both LHX8 protein and *Lhx8* mRNA expression in the inguinal adipose tissue (iAT) of C57/BL6 mice were significantly higher at postnatal day 6 (P6) than at postnatal day 56 (P56) (Figure 2A).

Based on this peak, we decided to abrogate LHX8 protein expression at P6 by transfecting one of the paired iAT depots with *Lhx8* small interfering RNA (siRNA). The contralateral iAT depot received an injection of transfection reagent only (serving as the control), and the two depots were subsequently compared at P10 (Figure 2B). This experimental design was chosen to ensure that both wild-type and *Lhx8* siRNA-transfected adipocytes were exposed to identical systemic cues, such as caloric intake and endocrine signals. Adipocyte-specific gene ablations are often generated by using a Cre-LoxP recombination under the control of the adiponectin promoter; however, we have dismissed this possibility due to the low adiponectin expression in the iAT of mice at P6 (Supplemental Figure S3H).

LHX8 protein expression was undetectable in the iAT following *Lhx8* siRNA transfection (Figure 2B), and the loss of LHX8 protein was uniform in the while iAT depot, as shown by scanning of the entire tissue sections (Supplemental Figure S4A). A similar loss of LHX8 was confirmed in in vitro cultured primary mouse adipocytes following *Lhx8* siRNA transfection (Figure 2B). The experimental loss of *Lhx8* resulted in adipose tissue expansion, as the *Lhx8* siRNA-transfected iAT depot was larger than its vehicle-treated counterpart (Figure 2C). This was accompanied by diminished UCP1 expression (Figure 2C) and an increase in adipocyte size (Figure 2D). Furthermore, mitochondrial content was lower in *Lhx8* siRNA-treated adipocytes than in controls and inflammatory cytokine expression was higher (Figure 2E,F). These cellular changes, which occurred without altering mitochondrial morphology (Supplemental Figure S4B,C), are consistent with increased lipogenesis and loss of thermogenic potential [29].

Additionally, beta-adrenergic stimulation of *Lhx8* siRNA-transfected mouse adipocytes failed to induce the expression of genes necessary for thermogenesis and lipolysis (Figure 2G), despite an increased *Adrb3*—encoding β3 adrenergic receptor, the major catecholamine receptor in mouse adipocytes—expression in *Lhx8* siRNA-transfected adipocytes (Supplemental Figure S4D), suggesting that the loss of LHX8 renders adipocytes resistant to catecholamines.

Using next-generation RNA sequencing (NGS), we identified differently expressed genes (DEGs) that were downregulated in *Lhx8* siRNA-transfected adipocytes compared with vehicle-treated controls. These DEGs belonged to a single gene network associated with three major pathways: interleukin-33 (IL-33) signaling, Th2 immune response, and MyoD1-controlled gene transcription (a hallmark of thermogenic adipocytes) (Figure 2H, Supplementary File S2).

This finding suggested a functional link to IL-33, a known potential inducer of thermogenic adipocyte development [37]. Consistent with this, we found that IL-33 triggered the expression of several thermogenic fat-associated genes in mouse primary adipocytes: *Ucp1*, *Ppargc1a*, *Myod1*, and *Dio2* (Figure 2I). Moreover, IL-33 enhanced the mRNA expression of *Adrb3* (encoding the major β-adrenergic receptor in mouse adipocytes) [38] (Figure 2I). The expression of genes associated with lipolysis was only moderately increased by IL-33 (Figure 2I). Treating mouse primary adipocytes with IL-33 reduced their lipid content and increased their UCP1 protein expression level (Figure 2J), providing evidence for an induced thermogenic phenotype.

### 3.5. Beta Adrenergic Stimulation Increases LHX8 Expression and Enhances Adipocyte IL-33 Synthesis in an LHX8-Depdendent Manner

**Figure 3:**
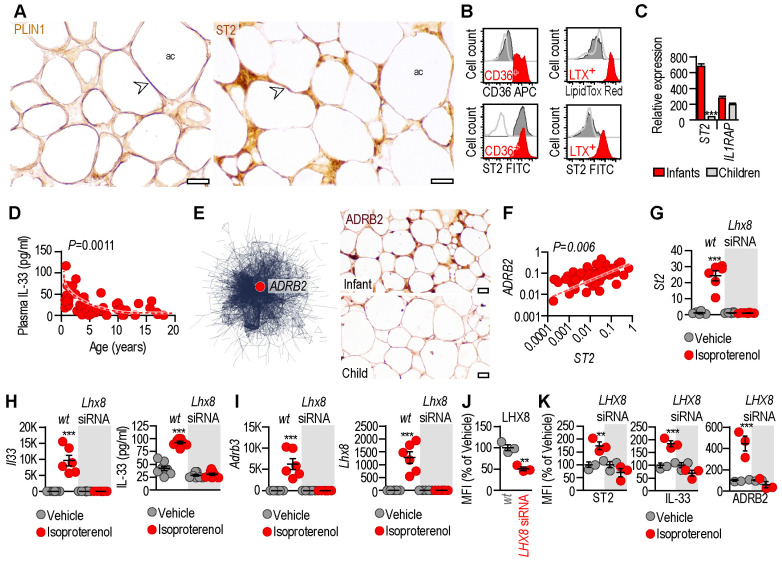
LHX8 is necessary for β-adrenergic signaling in adipocytes. (**A**) PLIN1 and ST2 immunostaining of human SAT, ac: adipocyte, arrowhead labels cell membranes, scale bar 30 μm. (**B**) Flow cytometry analysis of ST2^+^ cells in human adipose tissue. Preadipocytes were labeled with an antibody against CD36. Adipocytes were labeled with LipidTox Red (LTX). (**C**) Relative gene expression of *ST2* and interleukin-1 receptor accessory protein (*IL1RAP*) in infants (mean age 13.6 months, n = 3) and children (mean age 9 years, n = 3). Next-generation RNA sequencing, GEO accession number GSE271341. (**D**) Plasma IL-33 levels in children, each data point represents one patient. (**E**) Left: Protein–protein interaction map of gene products overrepresented in the SAT of infants compared with children. ADRB2 was a central hub within the overrepresented gene network. Right: Immunostaining of ADRB2 in the SAT of an infant (2 months of age) and a child (5 years of age), scale bar 30 μm. (**F**) Correlation of *ST2* and *ADRB2* expression in the SAT of children (n = 82). Spearman correlation analysis with Gaussian approximation. (**G**–**I**) Mouse primary adipocytes were transfected with non-silencing siRNA (*wt*) or *Lhx8* siRNA and treated with vehicle or isoproterenol for 48 h. (**G**) Effect of isoproterenol on the expression of *St2*. (**H**) Effect of isoproterenol on IL-33 mRNA expression and protein secretion. (**I**) Effect of isoproterenol on the expression of *Adrb3* and *Lhx8*. (**J**,**K**) Adipocytes of children were cultured in vitro and transfected with non-silencing siRNA (*wt*) or *LHX8* siRNA and treated with vehicle or isoproterenol for 4 h. (**J**) Mean fluorescence intensity (MFI) of LHX8 protein in adipocytes transfected with non-silencing siRNA (*wt*) or *LHX8* siRNA. (**K**) Effect of isoproterenol on the expression of ST2, IL-33 and ADRB2 proteins, expressed as relative MFI (% of vehicle). ** *p* < 0.01, *** *p* < 0.001 Student’s 2-tailed, unpaired *t*-test (**C**,**J**) or one-way ANOVA with Dunnett’s post hoc test (**G**–**I**,**K**).

We next investigated whether adipocytes express receptors for IL-33 and found that interleukin 1 receptor-like 1 (ST2), the major cell membrane receptor for IL-33, was expressed by human adipocytes (Figure 3A and Figure S5A). ST2 expression was associated with both CD36^+^ and lipid-laden adipocytes (Figure 3B), and it was present on both UCP1^−^ and UCP1^+^ adipocytes in human SAT (Supplemental Figure S5A). The expression level of *ST2* mRNA was positively associated with the mRNA levels of genes associated with thermogenesis in the SAT of children (Supplemental Figure S5B). Adipose tissue *ST2* expression peaked in perinatal life (Figure 3C). Correspondingly, the iAT of young mice at P6 expressed higher levels of *St2* mRNA than their adult counterparts (Supplemental Figure S5C). Interleukin-1 receptor accessory protein (IL1RAP), a co-receptor of ST2, was constitutively expressed in the SAT of human infants and children (Figure 3C).

Nuclear and cytosolic IL-33 expression was detectable in the adipose tissue of newborns and children, with ~30% of adipocytes expressing IL-33 during infancy and childhood (Supplemental Figure S5D,E). Furthermore, plasma IL-33 peaked after birth in humans (Figure 3D). By contrast, adipocytes of obese SAT expressed caspase 3, an enzyme known to inactivate IL-33 [39], and IL-33 was notably absent from obese human SAT (Supplemental Figure S5F). Murine adipocytes in the iAT constitutively expressed Il33 mRNA, and IL-33 expression was confined to the iAT (Supplemental Figure S5G,H).

Next, we aimed to define the upstream regulators of IL-33 and ST2 expression. We compared the mRNA expression profile of SAT specimens collected from infants and children. Overrepresented DEGs formed a gene network with the β2-adrenergic receptor (ADRB2) as a central hub in infants (Figure 3E). ADRB2 is the major β-adrenergic receptor in human SAT (Supplemental Figure S5I) and is the primary receptor that stimulates thermogenic gene expression in SAT in response to catecholamines [38,40]. Additionally, β-adrenergic stimulation triggers IL-33 synthesis in preadipocytes [37].

Adipocyte *ADRB2* mRNA and protein expression were detectable in human SAT during the perinatal period and childhood (Figure 3E and Figure S5I). Notably, *ST2* mRNA expression was positively associated with *ADRB2* mRNA expression in SAT throughout childhood (Figure 3F).

Beta adrenergic stimulation triggered the expression of both *St2* and *Il33* in mouse primary adipocytes in vitro (Figure 3G,H). These inductive effects were dependent on the presence of LHX8, as *Lhx8* siRNA transfection abrogated the effects of β-adrenergic stimulation (Figure 3G,H). Similarly, β-adrenergic stimulation of mouse adipocytes increased the expression of IL-33 protein in an LHX8-dependent manner (Figure 3H). The major receptor for catecholamines in mouse adipocytes is ADRB3 (Supplemental Figure S5I), and we found that β-adrenergic stimulation increased *Adrb3* mRNA expression (Figure 3I). This effect was likewise dependent on LHX8 (Figure 3I). Finally, β-adrenergic stimulation triggered *Lhx8* expression, establishing a positive feedback loop that was absent in the lack of LHX8 (Figure 3I). Similarly, human adipocytes transfected with *LHX8* siRNA failed to express ST2, IL-33, and ADRB2 in response to β-adrenergic stimulation (Figure 3J,K).

Both forskolin and bromo-cAMP mirrored the effect of β-adrenergic stimulation on the expression of *LHX8* (Supplemental Figure S6A). Conversely, blockage of cAMP response element-binding protein (CREB) signaling abolished the effect of β-adrenergic stimulation on LHX8 expression (Supplemental Figure S6A). In summary, β-adrenergic signaling increases LHX8 levels in adipocytes through the cAMP/CREB signaling pathway.

### 3.6. LHX8 Overexpression Increases Thermogenic Fat Development via IL-33 Signaling

**Figure 4:**
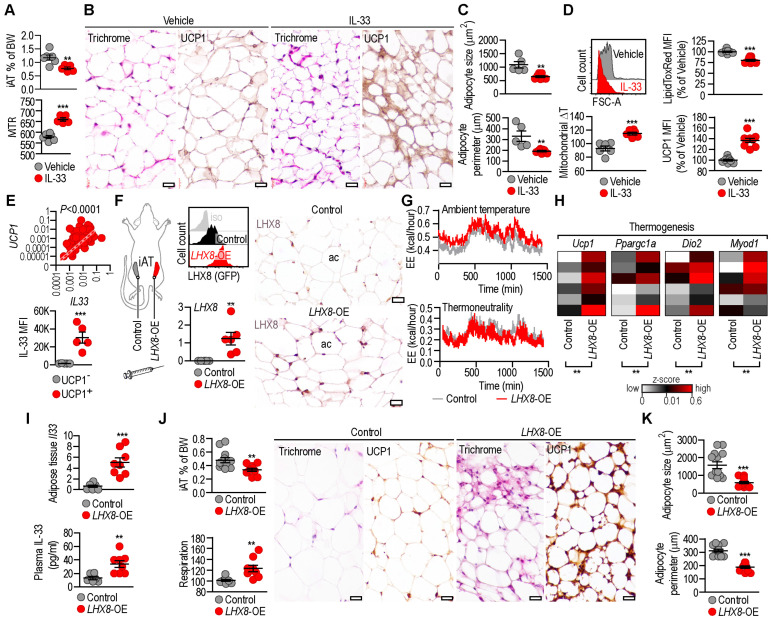
LHX8 overexpression increases adipose tissue browning. (**A**) C57/BL6 mice aged 8 weeks were treated with vehicle or IL-33 for 4 days. Percentage of iAT weight and body weight (iAT% of BW), and MitoTrackerRed (MTR) fluorescence intensity of the isolated adipocytes on day 4, n = 6 in each group. (**B**) Trichrome staining and UCP1 immunostaining of iAT on day 4, representative images from 6 animals in each group, scale bar 30 μm. (**C**) Adipocyte size and perimeter in iAT of vehicle- or IL-33-treated mice on day 4, n = 6 in each group. (**D**) Flow cytometry analysis of adipocytes from vehicle- or IL-33-treated mice. Forward scatter area (FSC-A), reflecting relative cell size; size scatter area (SSC-A), reflecting intracellular complexity. LipoToxRed staining and UCP1 expression of adipocytes. (**E**) *Top*: Correlation of *IL33* and *UCP1* mRNA expression in the adipose tissue of children aged 0.8–17 years. Spearman correlation analysis with Gaussian approximation. *Bottom*: IL-33 expression level in UCP1^−^ and UCP1^+^ adipocytes of children, measured by FACS analysis, n = 5, age 0.8–4 years. (**F**–**K**) Effects of overexpressing human LHX8 DNA in the iAT of C57/BL6 mice, aged 8 weeks. (**F**) Schematic illustration of LHX8 overexpression. Control: iAT was injected daily for 7 days with an empty vector. *LHX8*-OE: iAT was injected daily for 7 days with a human LHX8 DNA-containing vector. FACS analysis of LHX8 protein expression; qPCR measurement of *LHX8* mRNA; trichrome staining and UCP1 immunostaining of the iAT, ac: adipocyte, scale bar 30 μm. (**G**) Energy expenditure (EE) at ambient temperature and at thermoneutrality. (**H**) Expression levels of genes necessary for adipose tissue thermogenesis in iAT. (**I**) *Top*: Expression level of *Il33* in iAT. *Bottom*: plasma IL-33 levels. (**J**) Ratio of iAT weight and body weight (BW); NADH-DH activity of adipocytes; trichrome staining and UCP1 immunohistochemistry of iAT, scale bar 30 μm. (**K**) Adipocyte size and perimeter in the iAT, each data point represents one mouse, ** *p* < 0.01, *** *p* < 0.001 Student’s 2-tailed, unpaired *t*-test.

Treating mice with IL-33 led to several changes characteristic of a thermogenic switch: a decrease in iAT weight and adipocyte size, and an increased prevalence of UCP1^+^ adipocytes in the iAT (Figure 4A–C). Stimulation of ST2 with IL-33 specifically induced the phosphorylation of STAT6 in mouse primary adipocytes (Supplemental Figure S6B) and did not affect other potential interleukin-associated signal pathways (Supplemental Figure S6C). Furthermore, treating mouse adipocytes with IL-33 promoted the synthesis of IL-4 and inhibited IFNβ production (Supplemental Figure S6D).

Similarly, treating SAT-derived adipocytes of children with IL-33 in vitro resulted in a decrease in adipocyte size and lipid content, an increase in thermogenesis, and elevated UCP1 protein expression (Figure 4D). This was accompanied by STAT6 phosphorylation, increased ADRB2 and IL-13 expression, and decreased IFNβ expression (Supplemental Figure S6E–G).

Supporting a functional connection, adipose tissue *IL33* mRNA expression was positively associated with *UCP1* mRNA expression in children (Figure 4E), and UCP1^+^ human adipocytes strongly expressed IL-33 (Figure 4E). The latter finding suggests the presence of an autocrine IL-33 signaling loop in thermogenic UCP1^+^ adipocytes.

We next explored the effect of LHX8 overexpression on adipose tissue function. We transfected one of the iAT depots with *LHX8* mRNA to induce LHX8 protein overexpression (*LHX8*-OE) in adult mice (Figure 4F). LHX8 overexpression led to an increase in energy expenditure that was absent at thermoneutrality (Figure 4G), without inducing significant changes in other metabolic parameters (Supplementary File S1). This metabolic effect was concomitant with an increased expression of thermogenic genes in the iAT (Figure 4H). Moreover, LHX8 overexpression increased adipose tissue *Il33* mRNA expression and plasma IL-33 protein levels in mice (Figure 4I). In line with these findings, iAT size decreased, adipocyte mitochondrial activity increased, adipocyte size decreased, and UCP1 expression increased in response to LHX8 overexpression (Figure 4J,K).

Adipocytes of children with obesity displayed compromised LHX8 expression while maintaining sustained ADRB2 expression (Supplemental Figure S6H). Consistent with our finding that ADRB stimulation fails to induce thermogenesis in the absence of LHX8, ADRB stimulation did not induce UCP1 expression in LHX8-deficient adipocytes from children with obesity (Supplemental Figure S6I). Similarly, *LHX8* siRNA transfection abrogated the effect of isoproterenol on UCP1 expression in human adipocytes (Supplemental Figure S6I). However, IL-33 treatment of the LHX8-deficient human adipocytes was sufficient to induce UCP1 expression (Supplemental Figure S6J). Neutralization of IL-33 diminished the effectiveness of ADRB stimulation on UCP1 expression in LHX8-overexpressing human adipocytes (Supplemental Figure S6K).

**Figure 5:**
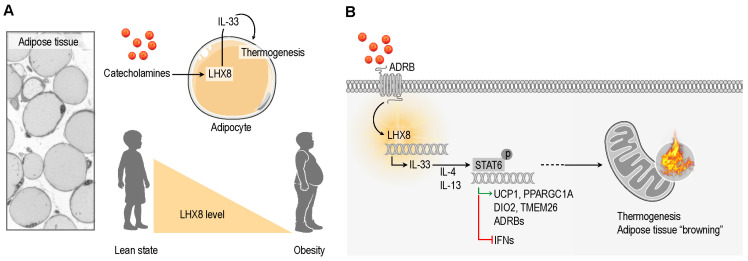
Role of the LHX8/IL-33 axis in human adipocytes. (**A**) Adipose tissue expresses LHX8 in adipocytes. Catecholamines stimulate LHX8 expression, which subsequently triggers IL-33 synthesis in adipocytes. Obesity compromises LHX8 expression. (**B**) ADRB activation enhances LHX8 expression through cAMP signaling. LHX8 promotes IL-33 expression, ultimately leading to Th2 cytokine synthesis and STAT6 phosphorylation within adipocytes. This cascade drives the expression of genes required for thermogenesis while simultaneously diminishing the expression of interferons (IFNs). The latter indirectly supports mitochondrial energy production and lipid catabolism. Collectively, LHX8 is necessary for catecholamine-induced dissipation of lipid-stored energy.

In summary, ADRB signaling increased LHX8 levels in mouse and human adipocytes, and LHX8 subsequently sustained IL-33 synthesis in both mouse and human adipocytes. IL-33 ultimately promoted the expression of adipocyte IL-4 (in mouse) and IL-13 (in humans), leading to STAT6 phosphorylation and a resulting increase in UCP1 expression (Figure S6L and Figure 5).

## 4. Discussion

Adipose tissue browning is the process by which thermogenic adipocytes develop within fat-storing adipose tissue depots, enhancing the capacity for the oxidative breakdown of stored lipids. A major physiological trigger of adipose tissue browning is the activation of β-adrenergic signaling by catecholamines. Obesity, however, often impairs the effectiveness of this catecholamine-induced browning [7,8,41]. This blunted response is a contributing factor to the challenges individuals with obesity face in achieving and maintaining weight loss.

Here we demonstrate that β-adrenergic stimulation of adipose tissue browning is dependent on the transcription factor LHX8, and this dependency is mediated by an autocrine signaling loop involving IL-33 within the adipocytes. Targeting this signaling axis could be a strategy to restore catecholamine sensitivity and promote effective thermogenesis in the context of obesity.

Adipose tissue browning predominantly occurs in SAT in humans, where catecholamine-induced thermogenesis is mediated by ADRB2 [38,40]. Contrastingly, browning in mice is chiefly mediated by ADRB3. In humans, ADRB3 is mostly expressed in visceral adipose deposits, such as the perirenal and omental fat [42,43,44]. Adipose tissue browning promotes both lipolysis and thermogenesis, which are closely related catabolic processes. Lipolysis provides free fatty acids for uncoupled oxidative phosphorylation in mitochondria, ultimately dissipating the energy stored in neutral lipids as heat. Catecholamines concurrently trigger both mechanisms; however, the induction of thermogenesis, specifically through the upregulation of UCP1 expression, may occur independently from lipolysis [45,46].

Impairment of ADRB2 signaling within the SAT can lead to a reduced sensitivity to catecholamines, a condition termed catecholamine resistance, abrogating both adipose tissue browning and the lipolytic breakdown of stored fat [41]. Catecholamine resistance may be a consequence of a sedentary lifestyle and can be a contributing factor to the development and maintenance of obesity [41]. Catecholamine resistance is also observed in the SAT depots of children with obesity [41,47,48], and may explain why the benefits of acute exercise in terms of reducing body fat are often attenuated or ineffective in this population [49]. Beyond catecholamine resistance, the overall thermogenic competence and capacity for adipose tissue browning are generally blunted in the obese adipose tissue of both children and adults [14,50]. Furthermore, a premature loss of thermogenic fat cells during infancy increases fat storage [36]. Restoring catecholamine responsiveness of adipocytes is hence key to reducing subcutaneous fat mass and promoting adipose tissue browning.

We found that catecholamine-induced adipose tissue browning was mediated by the LHX8/IL-33/ST2 signaling axis in both mouse and human adipocytes (Figure 5). Obesity reduced LHX8 expression, which ultimately compromised the β-adrenergic stimulation of IL-33 synthesis. IL-33, first identified in 2005, is an important, yet not fully understood cytokine [51]. It is typically stored in the nuclei of non-hematopoietic cells and released in response to cellular damage, functioning as an alarmin to elicit a Th2 immune response [52,53]. Consistent with this Th2 response, we have shown previously that STAT6 signaling—which is associated with an anti-inflammatory immune milieu in adipose tissue [54] —induces IL-33 expression [55]. Accordingly, the anti-inflammatory effects of IL-33 have been demonstrated within adipose tissue [56]. These anti-inflammatory actions indirectly improve systemic insulin sensitivity [57] and promote thermogenic adipocyte development in mice [58,59,60]. It should be noted, however, that this effect may decline with aging due to the loss of IL-33-responsive immune cells in aged adipose tissue [61].

We demonstrate that adipocytes synthesize IL-33, and that IL-33 exerts a direct autocrine effect on these cells, leading to the secretion of Th2 cytokines. This finding establishes that IL-33 triggers Th2 cytokine release not only from resident immune cells of the adipose tissue, but also directly from adipocytes themselves. Th2 cytokines are known to induce the expression of genes necessary for thermogenesis in adipocytes [62]. Furthermore, IL-33 was found to suppress the expression of inflammatory cytokines, including IFNβ. This anti-inflammatory action further supports adipose tissue browning [4,63,64,65], providing a mechanism by which this signaling axis may promote the loss of body fat.

Adipocyte-derived IL-33 directly stimulated UCP1 expression in both murine and human adipocytes. Crucially, this signaling was able to mitigate catecholamine resistance in adipocytes isolated from patients with obesity. This mechanism represents a novel therapeutic target for improving fat breakdown in obesity. Consistent with this function, loss of IL-33/ST2 signaling has been shown to exacerbate obesity in animal models, supporting the general consideration of IL-33 as a cytokine that improves obesity status [66].

However, despite its favorable Th2-associated profile, the role of IL-33 in obesity is complex and may include potential adverse effects. Although considered a Th2 cytokine, IL-33 may trigger the release of pro-inflammatory mediators in adipose tissue [67]. For instance, an excessive release of IL-33 from vascular endothelia has been observed in patients with morbid obesity [68]. This phenomenon could represent either a pro-resolving response to the chronic inflammation associated with obesity, or, alternatively, a contributing factor to the endothelial dysfunction frequently linked to the condition [68]. IL-33 levels are increased in the adipose tissue of patients with insulin resistance, and IL-33 may also inhibit glucose uptake by adipocytes [69]. This effect of IL-33, however, may also be interpreted as a compensatory mechanism to limit further lipogenesis by reducing the substrate (glucose) available for fat synthesis. Overall, IL-33 drives an immune response towards the Th2 phenotype within adipose tissue, a condition that may promote fat catabolism by reducing adipose tissue inflammation and increasing thermogenesis [60,66].

Unexpectedly, LHX8, a transcription factor primarily known for its role in gametogenesis, was identified as a positive regulator of IL-33 synthesis in adipocytes. Overexpression of LHX8 in adipocytes was found to promote IL-33 synthesis and subsequently drive adipocyte browning. We observed that adipocytes from obese individuals exhibited compromised IL-33 expression, and similarly, non-thermogenic UCP1^−^ adipocytes had negligible IL-33 levels. This confirms that the autocrine IL-33/ST2 signaling axis is intrinsically associated with thermogenic adipocytes and is diminished in obesity. Sympathetic nerve endings release IL-33, which promotes the expression of ADRBs [60]. Concurrently, catecholamines trigger IL-33 release from preadipocytes [37]. We also found that β-adrenergic stimulation of mouse and human adipocytes increased the expression of ADRB3 and ADRB2, respectively. Diminished LHX8 expression compromised these effects, indicating that LHX8 is necessary for the IL-33-induced expression of ADRBs. Therefore, the lack of LHX8 in obesity may directly account for the catecholamine resistance observed in adipocytes.

The restoration of LHX8 expression in the context of obesity represents a novel therapeutic strategy to enhance catecholamine sensitivity and promote weight loss. Targeting the adipose tissue via gene therapy is an active and promising field in the treatment of obesity [70,71,72]. Overexpressing LHX8 through targeted gene delivery to adipocytes could be a feasible intervention to resolve catecholamine resistance.

Our study identifies LHX8 as an essential mediator of adipocyte thermogenesis in response to catecholamines, providing new mechanistic insight into the regulation of energy expenditure. Therapeutic modulation of this pathway may offer new opportunities to mitigate catecholamine resistance, restore thermogenesis, and ultimately support efficient weight loss in individuals with obesity.

## 5. Conclusions

In the present study, we show that LHX8 expression is initiated early during human adipogenesis and is indispensable for driving catecholamine-induced thermogenesis. Critically, LHX8 overexpression was found to alleviate catecholamine resistance observed in adipocytes from obese individuals. Mechanistically, LHX8 stimulated adipocyte interleukin-33 (IL-33) synthesis in response to ADRB activation, leading to an increase in thermogenic gene expression in both human and mouse adipocytes. These findings position LHX8 as a promising novel therapeutic target to counteract catecholamine resistance in obese adipose tissue and ultimately facilitate weight loss.

## Data Availability

Raw NGS data are available in public repositories for secondary analysis (NIH GEO accession numbers GSE329222, GSE271341 and GSE274818). Unprocessed image files, FACS data and qPCR raw data are available through FigShare upon request; indirect calorimetry raw data are available in a Supplementary File of this article.

## Supplementary Materials

The following supporting information can be downloaded at: https://www.mdpi.com/article/10.3390/cells15111000/s1, Supplemental Figure S1: Anatomy and histology of the analyzed fat depots; Supplemental Figure S2: LHX8 immunostaining of human muscle and adipose tissue; Supplemental Figure S3: LHX8-expressing cells in human and murine adipose tissue; Supplemental Figure S4: Morphology of mouse adipocytes transfected with vehicle or *Lhx8* siRNA; Supplemental Figure S5: ST2, IL-33 and ADRB expression in human and murine adipose tissue; Supplemental Figure S6: LHX8 signaling in adipocytes; Supplemental Table S1: Information on patients donated fat biopsies in the study; Supplemental Table S2: Information on adipose tissue specimens collected post mortem; Supplemental Table S3: Human qPCR primer sequences used in the study; Supplemental Table S4: Mouse qPCR primer sequences used in the study; Supplemental Table S5: Antibodies used in the study; Supplementary File S1: Indirect calorimetry raw file; Supplementary File S2: Differently expressed genes following *Lhx8* siRNA transfection. Refs. [5,9,12,18,73,74,75,76,77,78,79,80] are cited in Supplementary Materials.
