# Supplementary material for: Lim Homeobox 8 Is Essential for Beta Adrenergic Stimulation of Thermogenesis in Human Adipocytes
Source: Cells. 2026 May 29;15(11):1000. doi: 10.3390/cells15111000 (PMC13256721; doi:10.3390/cells15111000)
Supplement: Supplementary file 1 [file cells-15-01000-s001.zip › Supplemental Information Gyurina-Radványi et al..pdf]

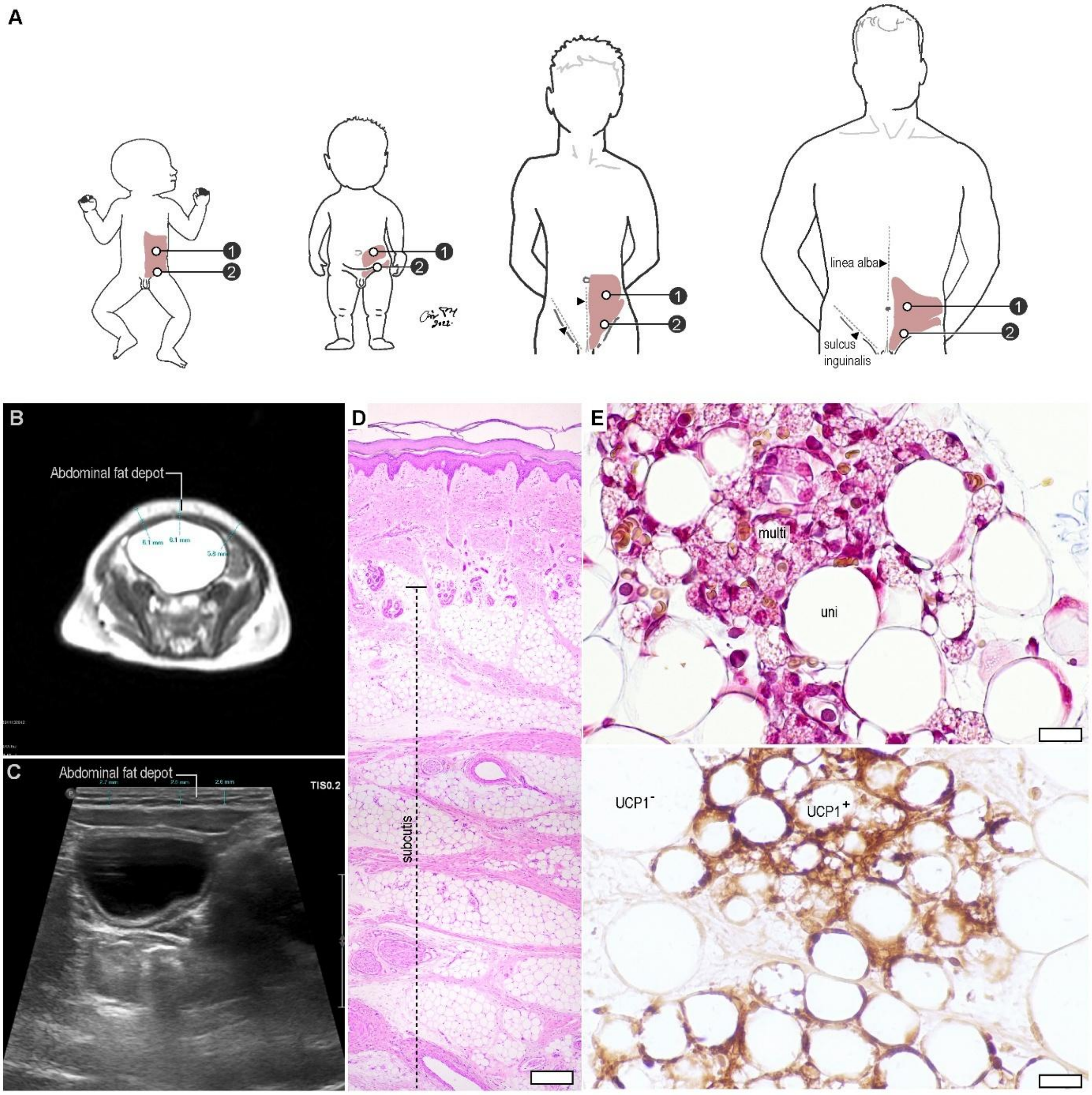

Supplemental Figure S1. Anatomy and histology of the analyzed fat depots
(**A**) Schematic representation of the localization of the analyzed fat depots in newborns, infants, children, and adolescents; ①: inguinal area of the abdominal subcutaneous fat depot, 64.3% of the analyzed biopsies were collected from this region; ②: umbilical and hypogastric regions of the abdominal subcutaneous fat depot, 35.7% of the analyzed biopsies were collected from this region. The inguinal region – typically the inguinal groove (sulcus inguinalis) – was the source of biopsies during orchidopexy and inguinal herniations. The umbilical and hypogastric regions were sources of biopsies during the surgical resolution of umbilical hernias or surgeries performed in the abdominal cavity, such as appendectomies. Visceral fat samples were collected from the mesoappendix and the large omentum during appendectomies. MRI (**B**) and ultrasound (**C**) images of the abdominal subcutaneous fat depot in infants (aged 2 and 5 months, representative images). (**D**) Abdominal wall cross section of a human newborn, aged 1 day, hematoxylin & eosin staining, scale bar 100 µm. (**E**) Trichrome staining and UCP1 immunostaining of the abdominal subcutaneous fat depot in a human infant, aged 5 days. multi: multilocular adipocytes, uni: unilocular adipocytes, scale bar 30 µm. In early postnatal life, the subcutaneous fat depot is rich in thermogenic adipocytes [1-5].

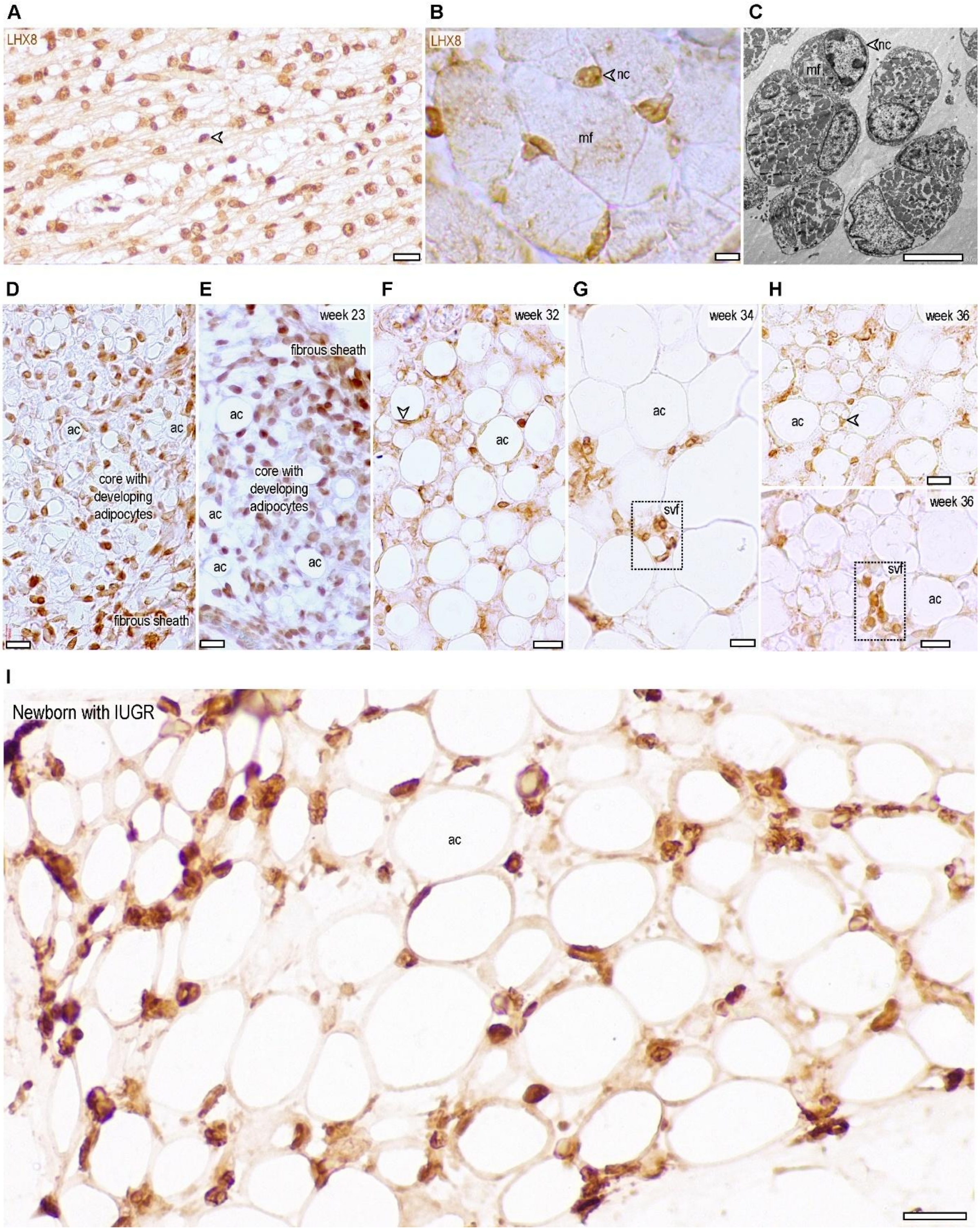

Supplemental Figure S2. LHX8 immunostaining of human muscle and adipose tissue LHX8 immunostaining of fetal spinal cord (**A**) and skeletal muscle (**B**) in the third trimester, scale bar 30 µm, nc: nucleus, mf: myofibers, representative images. (**C**) Transmission electron microscopy of fetal skeletal muscle, scale bar 2 µm, nc: nucleus, mf: myofiber, representative images. (**D–H**) LHX8 immunostaining of fetal adipose tissue of the abdominal wall in the second and third trimester, representative images (D: week 20, E: week 23, F: week 32, G: week 34, H: week 36), ac: adipocyte. svf: stromal vascular fraction, scale bar 30 µm. (**I**) LHX8 immunostaining in the abdominal subcutaneous adipose tissue of an infant born with intrauterine growth restriction (IUGR) and lived 5 days, ac: adipocyte, scale bar 30 µm.

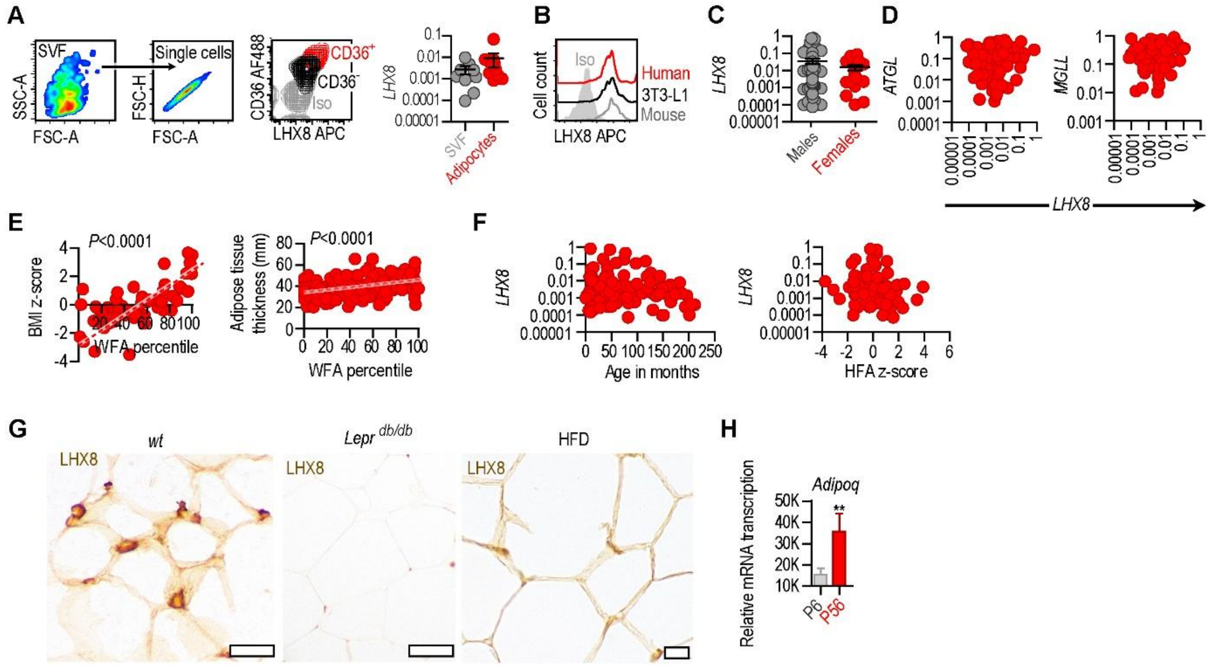

Supplemental Figure S3. LHX8-expressing cells in human and murine adipose tissue

(**A**) FACS analysis of the human adipose tissue stroma, SSC-A: side scatter area, FSC-A: forward scatter area, FSC-H: forward scatter height. Preadipocytes were defined as CD36+ cells. Using magnetic cell sorting, we separated stromal vascular cells (SVF) from mature adipocytes, as described elsewhere [6]. Expression of *LHX8* mRNA was measured by qPCR in SVF and mature adipocytes. Each data point represents one patient. (**B**) FACS analysis showing LHX8 protein expression in human adipocytes, mouse adipocytes isolated from inguinal adipose tissue (iAT), and 3T3-L1 preadipocytes. Representative histogram of three independent assays. (**C**) Comparison of adipose tissue *LHX8* mRNA levels in male and female patients. Each data point represents one patient. (**D**) Correlation of adipose tissue *LHX8*, *ATGL* and *MGLL* mRNA levels in abdominal subcutaneous adipose tissue specimens from children. Each data point represents one patient. (**E**) Correlation of weight-for-age (WFA) percentile and BMI *z*-score of children. Correlation of WFA percentile and abdominal subcutaneous adipose tissue thickness. Adipose tissue thickness was measured with ultrasonography. Each data point represents one patient. (**F**) Age, height-for-age (HFA) *z*-scores and adipose tissue *LHX8* mRNA levels in children. (**G**) LHX8 immunostaining of the iAT of wild-type (*wt*), leptin-resistant obese ($Lepr^{db/db}$), and high-fat diet (HFD)-fed obese mice at 16 weeks of age, scale bar 30 μm. Representative images. (**H**) Relative transcription of *Adipoq*, encoding adiponectin, in the iAT of mice at P6 and P56. Secondary analysis of NGS data from our previous studies [7,8]. ***P*<0.01 Student's 2tailed, unpaired *t*-test

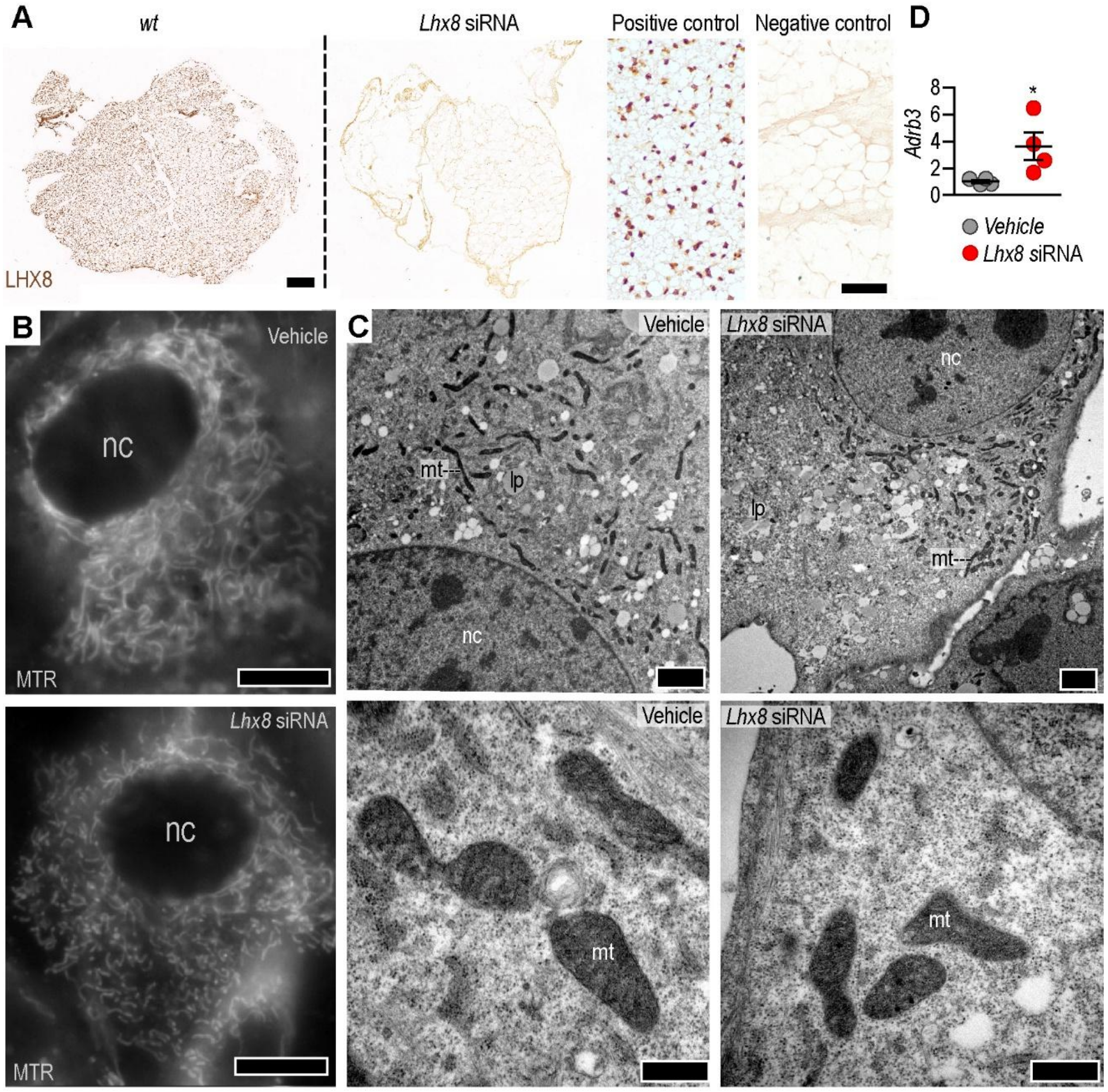

Supplemental Figure S4. Morphology of mouse adipocytes transfected with vehicle or *Lhx8* siRNA

(**A**) LHX8 immunostaining of iAT depots transfected with vehicle (*wt*) or *Lhx8* siRNA. Entire tissue sections have been scanned with a slide scanner. Scale bar 0.5 cm. As a positive control of the LHX8 immunostaining we used mouse brown adipose tissue, where LHX8 is expressed [9-12]. As a negative control we omitted primary antibody during the staining procedure. Scale bar 50 µm. (**B**) MitoTrackerRed (MTR) labeling of mouse adipocytes transfected with vehicle (*wt*) or *Lhx8* siRNA. Scale bar 10 µm. nc: nucleus (**C**) Transmission electron microscopy of mouse adipocytes transfected with vehicle (*wt*) or *Lhx8* siRNA. mt: mitochondria, nc: nucleus, scale bar 10 µm (*Top*) and 2 µm (*Bottom*). (**D**) Relative mRNA expression of *Adrb3* in the iAT transfected with vehicle (*wt*) or *Lhx8* siRNA. *P<0.05, Student's2-tailed, unpaired t-test.

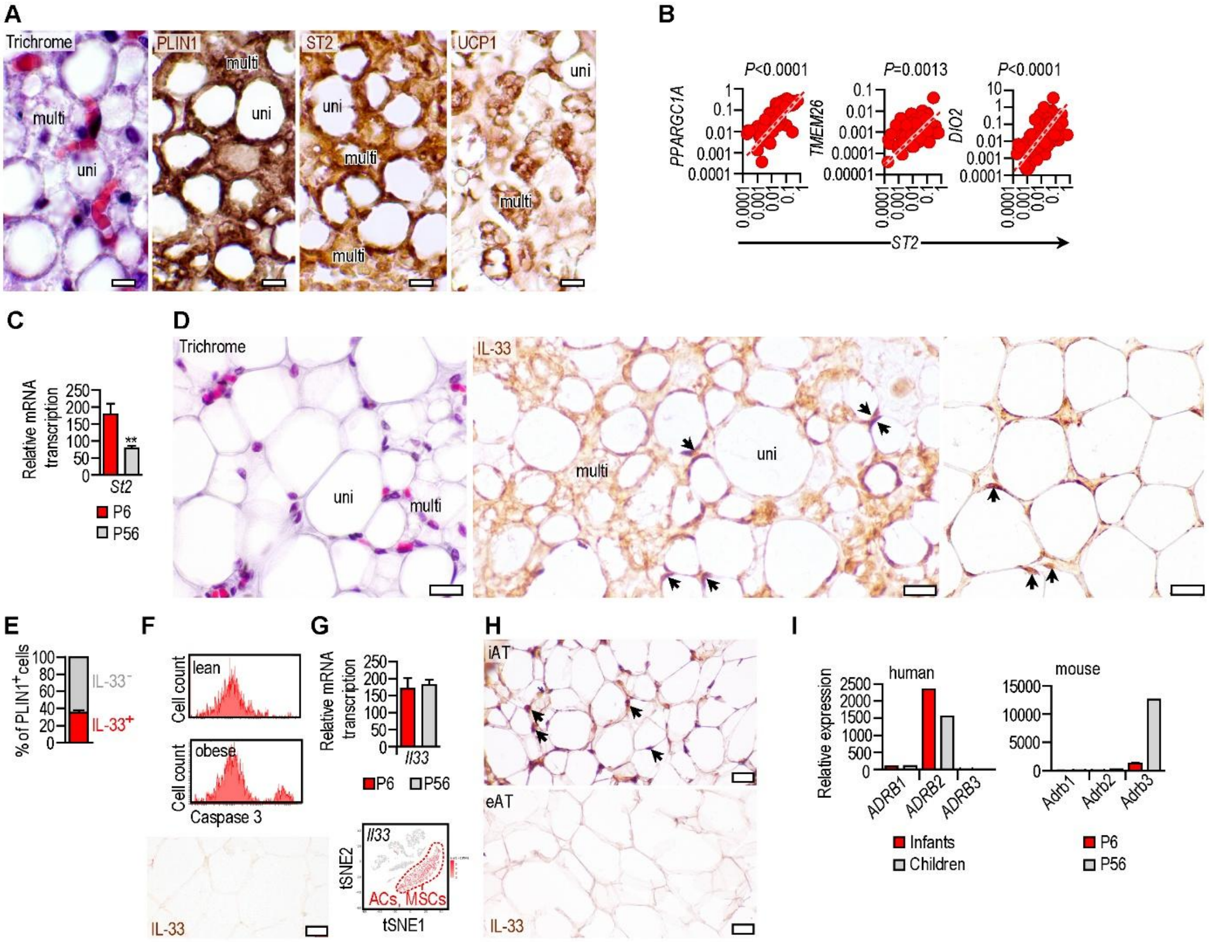

Supplemental Figure S5. ST2, IL-33 and ADRB expression in human and murine adipose tissue (**A**) SAT in a human fetus at gestational week 32. Masson’s trichrome staining, PLIN1, ST2 and UCP1 immunostaining, uni: unilocular adipocyte, multi: multilocular adipocyte, scale bar 5 µm. (**B**) Correlation of *ST2* levels with the expression levels of thermogenic genes (*PPARGC1A, TMEM26* and *DIO2*) in the adipose tissue of children. Correlation analysis with Gaussian approximation. (**C**) Expression level of *St2* mRNA in the iAT of mice at postnatal day 6 and 56 (P6 and P56, respectively). Secondary analysis of NGS data from our previous study, n=3 in each group [7]. (**D**) Trichrome staining and IL-33 immunostaining of the abdominal subcutaneous adipose tissue of a newborn and an infant; arrows label adipocyte nuclei, uni: unilocular adipocyte, multi: multilocular adipocyte, scale bar 30 µm. (**E**) Percentage of IL-33$^+$ adipocytes in the abdominal subcutaneous adipose tissue. Adipocytes were isolated from biopsies of five donors (0.3 years–14 years of age), and IL-33 expression was measured in PLIN1$^+$ cells by FACS. (**F**) *Top:* FACS histogram showing caspase 3-expressing adipocyte populations. *Bottom:* IL-33 immunostaining in the abdominal subcutaneous adipose tissue of a child living with obesity (WFA percentile 86), scale bar 30 µm. (**G**) *Top:* Relative mRNA expression of *Il33* in the iAT of mice at P6 and P56. Secondary analysis of NGS data from our previous study, n=3 in each group [7]. *Bottom:* Tabula Muris data showing the expression of *Il33* mRNA in adipocytes and mesenchymal stem cells (ACs, MSCs). (**H**) Immunostaining of IL-33 in mouse iAT and epididymal adipose tissue (eAT). Arrows label adipocyte nuclei, scale 30 µm. (**I**) Relative expression of *ADRB1*, *ADRB2*, and *ADRB3* in the subcutaneous adipose tissue of infants and children (n=3 in each age group); *Adrb1*, *Adrb2*, and *Adrb3* in the iAT of mice at P6 and P56. Secondary analysis of NGS data from our previous studies [7,8]. ***P*<0.01, Student’s 2-tailed, unpaired *t*-test.

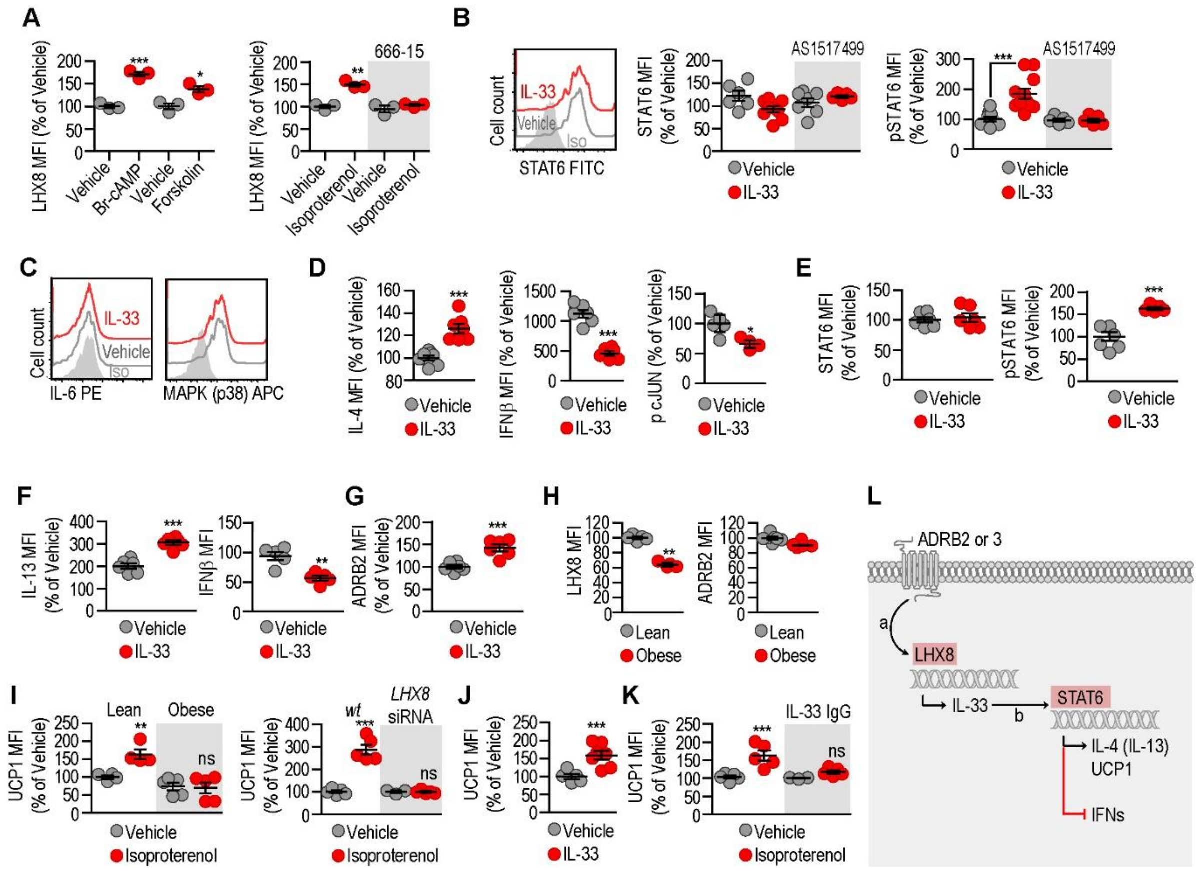

Supplemental Figure S6. LHX8 signaling in adipocytes
(**A**) *Left:* Mean fluorescence intensity (MFI) of LHX8 protein in mouse adipocytes, measured by FACS. Cells were cultured *in vitro* and treated with 500 µM 8-bromo-cAMP (Br-cAMP) or 50 µM forskolin for 48 h. *Right:* MFI of LHX8 protein in mouse adipocytes treated with vehicle or 100 µM isoproterenol for 48 h. In a complementary assay, the cells were incubated with CREB inhibitor 666-15. (**B**) FACS histogram showing STAT6 levels in mouse adipocytes treated *in vitro* with vehicle or 30 ng/ml IL-33 for 4 h. Iso: isotype control. MFI of STAT6 and phosphorylated STAT6 (pSTAT6) were measured. AS1517499: STAT6 inhibitor. (**C**) MFI and representative FACS histograms of IL-6 and MAPK (p38) in mouse adipocytes treated *in vitro* with vehicle or 30 ng/ml L-33 for 4 h. (**D**) MFI of IL-4, IFNβ and phosphorylated c-JUN (p cJUN) in mouse adipocytes treated with vehicle or 30 ng/ml IL-33 for 4 h. (**E**) MFI of STAT6 and pSTAT6 in human adipocytes treated *in vitro* with vehicle or 30 ng/ml L-33 for 4 h. Adipocytes were harvested from 6 donors (age 0.5–8 years), each data point represents one patient. (**F**) IL-13 and IFNβ MFI in human adipocytes treated *in vitro* with 30 ng/ml IL-33 for 4 h. Adipocytes were harvested from 6 donors. (**G**) ADRB2 MFI in human adipocytes treated *in vitro* with 30 ng/ml IL-33 for 4 h. Adipocytes were harvested from 6 donors, each data point represents one patient. (**H**) LHX8 and ADRB2 MFI in adipocytes of lean (WFA below 50 WFA percentile, age 2-8 years) and obese children (WFA above 90 percentile, age 4–8 years). (**I**) UCP1 MFI in human adipocytes treated *in vitro* with 100 µM isoproterenol for 4 h. *Left:* Adipocytes were harvested from 5 lean and 4 obese donors, age 4–8 years. *Right:* Adipocytes were transfected with non-silencing siRNA (*wt*) or LHX8 siRNA. (**J**) UCP1 MFI in human adipocytes treated *in vitro* with 30 ng/ml IL-33 for 4 h. Adipocytes were harvested from obese donors, age 6–8 years. (**K**) UCP1 MFI in human adipocytes overexpressing *LHX8*, and treated with vehicle or isoproterenol for 4 h. IL-33 IgG: neutralizing antibody against IL-33, 0.1 µg/ml. ns: nonsignificant, *P<0.05, **P<0.01, ***P<0.001 Student's unpaired *t*-test. (**L**) Schematic illustration of the ADRB/LHX8/IL-33 signaling axis: a: cAMP-dependent upregulation of LHX8 expression, b: IL-33 mediated upregulation of IL-4/IL-13, and inhibition of interferon response genes (IFNs).

Supplemental Table S1. Information on patients donated fat biopsies in the study

Sex	Fat depot	Age (months)	Height (cm)	Weight (kg)	BMI	WFA %
m	abdominal subcutaneous	2	49	3.20	13.33	0.12
m	abdominal subcutaneous	6	66	8.20	18.82	62.62
m	abdominal subcutaneous	7	74	8.80	16.07	65.50
m	abdominal subcutaneous	8	69	7.60	15.96	8.66
m	abdominal subcutaneous	8	69	9.17	19.26	61.73
m	abdominal subcutaneous	9	76	13.00	22.51	99.82
m	abdominal subcutaneous	9	70	7.00	14.29	0.53
m	abdominal subcutaneous	9	69	7.80	16.38	5.94
m	abdominal subcutaneous	9	75	9.60	17.07	61.84
m	abdominal subcutaneous	10	70	9.00	18.37	26.33
m	abdominal subcutaneous	10	74	9.30	16.98	36.80
m	abdominal subcutaneous	10	74	8.50	15.52	12.45
m	abdominal subcutaneous	11	71	9.00	17.85	16.94
m	abdominal subcutaneous	12	76	10.50	18.18	56.55
m	abdominal subcutaneous	12	79	10.50	16.82	56.55
m	abdominal subcutaneous	12	78	10.00	16.44	39.07
m	abdominal subcutaneous	12	75	9.00	16.00	10.63
m	abdominal subcutaneous	12	74	7.90	14.43	0.67
m	abdominal subcutaneous	12	77	12.00	20.24	91.25
m	abdominal subcutaneous	12	77	10.50	17.71	56.55
m	abdominal subcutaneous	12	76	9.50	16.45	22.76
m	abdominal subcutaneous	13	74	10.00	18.26	29.86
m	abdominal subcutaneous	13	87	12.00	15.85	86.89
m	abdominal subcutaneous	13	81	11.30	17.22	72.01
m	abdominal subcutaneous	13	85	12.50	17.30	93.10
m	abdominal subcutaneous	14	72	7.00	13.50	0.00

m	abdominal subcutaneous	14	79	9.50	15.22	10.89
m	abdominal subcutaneous	15	81	9.00	13.72	2.55
m	abdominal subcutaneous	15	82	11.50	17.10	62.85
m	abdominal subcutaneous	15	82	11.40	16.95	59.80
m	abdominal subcutaneous	16	84	11.00	15.59	39.72
m	abdominal subcutaneous	16	83	11.60	16.84	59.00
m	abdominal subcutaneous	21	87	16.00	21.14	99.26
m	abdominal subcutaneous	23	85	13.00	17.99	63.40
m	abdominal subcutaneous	23	89	11.50	14.52	21.27
f	abdominal subcutaneous	24	87	11.00	14.53	18.88
m	abdominal subcutaneous	24	84	11.00	15.59	9.23
m	abdominal subcutaneous	26	87	12.00	15.85	23.89
m	abdominal subcutaneous	27	98	20.00	20.82	99.99
m	abdominal subcutaneous	27	85	11.40	15.78	9.74
m	abdominal subcutaneous	27	92	15.70	18.55	94.91
m	abdominal subcutaneous	31	80	11.00	17.19	3.60
m	abdominal subcutaneous	34	95	13.50	14.96	35.90
m	abdominal subcutaneous	34	90	14.00	17.28	48.85
f	abdominal subcutaneous	37	104	17.00	15.72	92.75
f	mesoappendix	37	104	17.00	15.72	92.75
m	abdominal subcutaneous	38	92	13.00	15.36	13.85
m	abdominal subcutaneous	38	86	10.00	13.52	0.01
m	abdominal subcutaneous	38	99	15.00	15.30	58.83
m	abdominal subcutaneous	38	84	12.00	17.01	9.30
m	abdominal subcutaneous	38	103	18.00	16.97	95.73
f	abdominal subcutaneous	39	99	15.50	15.81	73.17
m	abdominal subcutaneous	40	100	16.00	16.00	72.38
f	abdominal subcutaneous	40	92	14.00	16.54	38.97

m	abdominal subcutaneous	41	100	15.00	15.00	48.08
m	abdominal subcutaneous	41	113	15.00	11.75	48.08
f	abdominal subcutaneous	42	103	15.00	14.14	54.21
m	abdominal subcutaneous	42	98	14.50	15.10	32.96
f	abdominal subcutaneous	42	104	15.50	14.33	64.01
m	abdominal subcutaneous	42	90	12.00	14.81	1.12
m	abdominal subcutaneous	43	106	16.00	14.24	62.74
m	abdominal subcutaneous	44	100	14.00	14.00	17.13
m	abdominal subcutaneous	44	100	16.00	16.00	59.31
m	abdominal subcutaneous	44	98	15.00	15.62	37.43
m	abdominal subcutaneous	45	105	16.00	14.51	55.81
m	abdominal subcutaneous	46	110	18.00	14.88	84.12
m	abdominal subcutaneous	46	100	14.00	14.00	12.79
m	abdominal subcutaneous	46	102	13.00	12.50	3.13
m	abdominal subcutaneous	47	104	17.00	15.72	67.96
m	abdominal subcutaneous	47	104	20.00	18.49	95.32
m	abdominal subcutaneous	50	104	16.00	14.79	38.31
m	abdominal subcutaneous	50	103	17.00	16.02	58.14
m	abdominal subcutaneous	50	109	19.00	15.99	85.48
m	abdominal subcutaneous	51	99	16.00	16.32	35.00
m	abdominal subcutaneous	51	111	19.50	15.83	87.70
f	abdominal subcutaneous	53	113	20.00	15.66	88.62
m	abdominal subcutaneous	54	107	19.00	16.60	76.57
m	abdominal subcutaneous	56	104	16.00	14.79	20.67
m	abdominal subcutaneous	56	105	16.50	14.97	29.00
f	abdominal subcutaneous	57	112	20.00	15.94	82.33
m	abdominal subcutaneous	57	110	18.50	15.29	61.13
m	abdominal subcutaneous	58	118	23.00	16.52	95.51

m	abdominal subcutaneous	59	110	19.00	15.70	62.74
m	abdominal subcutaneous	59	95	14.60	16.18	2.75
m	abdominal subcutaneous	60	111	19.00	15.42	59.75
m	abdominal subcutaneous	63	118	16.50	11.85	12.93
m	abdominal subcutaneous	65	117	32.00	23.38	99.84
f	abdominal subcutaneous	67	122	25.00	16.80	93.53
m	abdominal subcutaneous	71	112	18.00	14.35	15.73
m	abdominal subcutaneous	73	116	19.00	14.12	23.87
m	abdominal subcutaneous	73	114	16.50	12.70	2.73
m	abdominal subcutaneous	73	121	24.50	16.73	85.38
m	abdominal subcutaneous	74	110	15.50	12.81	0.46
m	abdominal subcutaneous	74	119	19.00	13.42	21.73
m	abdominal subcutaneous	74	104	14.00	12.94	0.01
m	abdominal subcutaneous	74	110	27.00	22.31	93.91
m	abdominal subcutaneous	75	127	26.00	16.12	89.94
m	abdominal subcutaneous	77	120	19.80	13.75	25.33
m	abdominal subcutaneous	78	115	20.00	15.12	25.66
m	abdominal subcutaneous	80	114	21.00	16.16	33.85
m	abdominal subcutaneous	80	117	22.00	16.07	46.79
m	abdominal subcutaneous	82	129	55.00	33.05	99.99
m	abdominal subcutaneous	83	124	27.00	17.56	84.84
m	abdominal subcutaneous	83	105	22.00	19.95	39.41
m	abdominal subcutaneous	84	128	34.00	20.75	98.21
m	abdominal subcutaneous	86	122	23.50	15.79	50.26
m	abdominal subcutaneous	89	145	31.00	14.74	92.33
m	mesoappendix	89	145	31.00	14.74	92.33
m	abdominal subcutaneous	89	122	23.00	15.45	37.37
m	abdominal subcutaneous	93	133	23.00	13.00	28.55

m	abdominal subcutaneous	96	128	26.00	15.87	53.54
m	abdominal subcutaneous	97	118	25.00	17.95	41.17
m	abdominal subcutaneous	99	143	39.00	19.07	97.29
m	abdominal subcutaneous	99	135	27.50	15.09	60.53
m	abdominal subcutaneous	101	137	29.50	15.72	71.32
f	abdominal subcutaneous	102	135	26.00	14.27	39.42
m	abdominal subcutaneous	105	142	42.00	20.83	97.36
f	abdominal subcutaneous	107	144	29.00	13.99	52.32
f	mesoappendix	107	144	29.00	13.99	52.32
m	abdominal subcutaneous	107	128	25.00	15.26	20.76
m	abdominal subcutaneous	109	140	29.00	14.80	51.53
m	abdominal subcutaneous	110	140	31.00	15.82	64.23
m	abdominal subcutaneous	116	143	38.00	18.58	85.67
m	abdominal subcutaneous	118	118	24.00	17.24	3.65
m	abdominal subcutaneous	119	130	29.00	17.16	30.26
f	abdominal subcutaneous	120	140	31.00	15.82	37.81
m	abdominal subcutaneous	120	140	30.00	15.31	35.79
m	mesoappendix	120	140	30.00	15.31	35.79
m	abdominal subcutaneous	125	130	29.00	17.16	19.58
m	abdominal subcutaneous	126	148	36.00	16.44	62.72
m	abdominal subcutaneous	127	155	57.00	23.73	98.25
m	abdominal subcutaneous	129	139	37.00	19.15	62.18
m	abdominal subcutaneous	130	151	43.00	18.86	83.16
m	abdominal subcutaneous	137	155	50.00	20.81	89.97
f	abdominal subcutaneous	141	135	28.00	15.36	1.69
m	abdominal subcutaneous	144	146	30.00	14.07	4.15
m	abdominal subcutaneous	146	135	35.00	19.20	18.44
m	mesoappendix	146	135	35.00	19.20	18.44
m	abdominal subcutaneous	148	157	50.00	20.28	79.17

m	abdominal subcutaneous	149	158	61.00	24.44	94.42
m	mesoappendix	149	158	61.00	24.44	94.42
m	abdominal subcutaneous	151	159	73.00	28.88	98.53
m	mesoappendix	151	159	73.00	28.88	98.53
f	abdominal subcutaneous	153	145	40.00	19.02	27.40
f	mesoappendix	153	145	40.00	19.02	27.40
f	mesoappendix	155	157	55.00	22.31	81.07
m	abdominal subcutaneous	157	158	47.00	18.83	54.17
m	abdominal subcutaneous	159	147	36.00	16.66	6.90
m	abdominal subcutaneous	163	168	60.00	21.26	84.18
m	abdominal subcutaneous	163	165	83.00	30.49	98.98
m	mesoappendix	163	168	60.00	21.26	84.18
m	mesoappendix	163	165	83.00	30.49	98.98
m	abdominal subcutaneous	165	177	50.00	15.96	51.47
m	abdominal subcutaneous	173	176	54.00	17.43	52.96
f	abdominal subcutaneous	176	157	66.40	26.94	88.74
m	abdominal subcutaneous	182	168	55.00	19.49	41.63
f	abdominal subcutaneous	185	156	70.00	28.76	90.61
m	abdominal subcutaneous	187	161	52.50	20.25	24.19
m	abdominal subcutaneous	191	171	55.00	18.81	28.19
f	abdominal subcutaneous	192	159	60.00	23.73	72.07
m	abdominal subcutaneous	193	164	95.00	35.32	98.40
m	mesoappendix	193	175	55.00	17.96	25.62
f	abdominal subcutaneous	195	165	72.00	26.45	91.16
f	mesoappendix	195	165	72.00	26.45	91.16
m	abdominal subcutaneous	196	180	65.00	20.06	59.82
m	abdominal subcutaneous	201	162	53.00	20.20	11.41
m	abdominal subcutaneous	205	183	87.00	25.98	93.88
f	abdominal subcutaneous	209	161	78.00	30.09	94.11
f	mesoappendix	209	161	78.00	30.09	94.11
m	abdominal subcutaneous	211	165	98.00	36.00	97.57

m: male. f: female, BMI: body mass index, WFA: weight-for-age

Supplemental Table S2. Information on adipose tissue specimens collected *post mortem*

Sex	Gestational age (weeks)	Age at death	Cause of fatality/abortion
m	17 weeks	in utero	trisomy 21
non-defined	17 weeks	in utero	trisomy 21
m	19 weeks	in utero	phocomelia
f	19 weeks	in utero	spina bifida
m	19 weeks	in utero	trisomy 21
m	20 weeks	in utero	trisomy 21
m	20 weeks	in utero	cleft lip and palate
f	20 weeks	in utero	heart defect
m	20 weeks	in utero	trisomy 21
m	21 weeks	in utero	cerebral ventriculomegaly
f	22 weeks	in utero	placental abruption
m	23 weeks	19 hours postnatal	infant respiratory distress syndrome
f	23 weeks	3 days postnatal	infant respiratory distress syndrome
m	30 weeks	in utero	placental abruption
m	32 weeks	in utero	placental abruption
f	33 weeks	3 days postnatal	infant respiratory distress syndrome
m	33 weeks	2.5 days postnatal	intracranial bleeding
m	34 weeks	in utero	intrauterine asphyxia
f	36 weeks	in utero	placental abruption
m	38 weeks	in utero	placental abruption
m		7 years	acute lymphoid leukemia
m		8 years	cystic fibrosis
m		39 years	pulmonary embolism
f		70 years	pulmonary embolism

m: male. f: female

Supplemental Table S3. Human qPCR primer sequences used in the study (Merck)

ACTB	fw	CACCSTTGGCAATGAGCGGTTC
	rev	AGGTCTTTGCGGATGTCCACGT
GAPDH	fw	GTCTCCTCTGACTTCAACAGCG
	rev	ACCACCCTGTTGCTGTAGCCAA
LHX8	fw	GGACCAGCTTTACAGCAGATCAG
	rev	CGTCTGCTCAAGCCTGTCCTTT
UCP1	fw	AGTTCCTCACCGCAGGGAAAGA
	rev	GTAGCGAGGTTTGATTCCGTGG
PPARGC1A	fw	CCAAAGGATGCGCTCTCGTTCA
	rev	CGGTGTCTGTAGTGGCTTGACT
MYOD1	fw	CTCCAACTGCTCCGACGGCAT
	rev	ACAGGCAGTCTAGGCTCGACAC
DIO2	fw	TTGAGCCGCTCCAAGTCCACTC
	rev	CTGTACTGGAGACATGCACCAC
IL33	fw	GCCTGTCAACAGCAGTCTACTG
	rev	TGTGCTTAGAGAAGCAAGATACTC
ST2	fw	CTCTGTTTCCAGTAATCGGAGCC
	rev	GCAGCCAAGAACTGAGTGCCTT
TMEM26	fw	GCAGTTTCCACTTGACCTGGCA
	rev	GAAGACGCTGATTCCGATGTTCC
ADRB2	fw	TACCAGAGCCTGCTGACCAAGA
	rev	AGTCACAGCAGGTCTCATTGGC
ATGL	fw	CCCACTTCAACTCCAAGGACGA
	rev	GCAGGTTGTCTGAAATGCCACC
MGLL	fw	GGCATGGTACTCATTTCGCCTC
	rev	GTTTGGCAGCACAAGGTTGAGC

Supplemental Table S4. Mouse qPCR primer sequences used in the study (Merck)

Actb	fw	GCACCAGGGTGTGATGGTG
	rev	CCAGATCTTCTCCATGTCGTCC
Gapdh	fw	TTGACGTGCCGCCTGGAGAAA
	rev	AGTGTAGCCCAAGATGCCCTTCAG
Lhx8	fw	AGCACAGTTCGCTCAGGACAAC
	rev	GCTGAGGAAGAATGGTTGGGAC
Ucp1	fw	CCTGCCTCTCTCGGAAACAA
	rev	CTGTAGGCTGCCCAATGAAC
Ppargc1a	fw	GACTCAGTGTCACCAACCGAAA
	rev	TGAACGAGAGCGCATCCTT
Myod1	fw	GCACTACAGTGGCGACTCAGAT
	rev	TAGTAGGCGGTGTCGTAGCCAT
Dio2	fw	GTCCGCAAATGACCCCTTT
	rev	CCCACCCACTCTCTGACTTTC
Il33	fw	CTACTGCATGAGACTCCGTTCTG
	rev	AGAATCCCGTGGATAGGCAGAG
SIl1rl1	fw	GGATTGAGGTTGCTCTGTTCTGG
	rev	TCGGGCAGAGTGTGGTGAACAA
Adrb3	fw	GAGCGACTACAAACCGTCACCA
	rev	TGGAAGTCCAGAACTCGCACCA
Atgl	fw	GGAACCAAAGGACCTGATGACC
	rev	ACATCAGGCAGCCACTCCAACA
Mgll	fw	GACACCATCCAGAAGGACTACC
	rev	GATTGGCAAGGACCAGAGGTGA
Tnfa	fw	TGCCTATGTCTCAGCCTCTTC
	rev	GAGGCCATTTGGGAACTTCT
Ifna	fw	TGAAGGACAGGAAGGACTTTG
	rev	GAATGAGTCTAGGAGGGTTGT

Supplemental Table S5. Antibodies used in the study (h: human; m: mouse)

**Target**	**Cat. No.**	**IgG type, dilution, source**
h/m LHX8	PA5-102563	rabbit polyclonal, 1:200 ThermoFisher Scientific, Rockford, IL
h/m UCP1	PA1-24894	rabbit polyclonal, 1:200 ThermoFisher Scientific, Rockford, IL
h/m perilipin 1 (PLIN1)	690156S	mouse monoclonal, 1:200 ThermoFisher Scientific, Rockford, IL
h/m ST2	PA5-20077	rabbit polyclonal, 1:200 ThermoFisher Scientific, Rockford, IL
h/m ADRB2	PA5-14117	rabbit polyclonal, 1:200 ThermoFisher Scientific, Rockford, IL
h CD36	14-0369-82	mouse polyclonal, 1:200 ThermoFisher Scientific, Rockford, IL
h CD36 APC	MA1-10210	mouse monoclonal, 10 µl/test ThermoFisher Scientific, Rockford, IL
h/m IL-33	12372-1-AP	rabbit polyclonal, 1:400 Proteintech Europe, Manchester, UK
h IL-13 FITC	BMS133FI	mouse monoclonal, 10 µl/test ThermoFisher Scientific, Rockford, IL
m IL-4	12-7041-81	mouse monoclonal, 1:200 ThermoFisher Scientific, Rockford, IL
h IFNβ	BMS1044FI	mouse monoclonal, 1:200 ThermoFisher Scientific, Rockford, IL
h IL-6 APC	MQ2-13A5	rat monoclonal, 5 µL/test eBioscience, San Diego, CA
h/m STAT6	#9362	rabbit polyclonal, 1:100 Cell Signaling Technology, Danvers, MA
h p-STAT6	#9361	rabbit polyclonal, 1:100 Cell Signaling Technology, Danvers, MA
h/m p38	MA5-37229	rabbit recombinant monoclonal, 1:200 ThermoFisher Scientific, Rockford, IL
h/m phospho-c-JUN	MA5-52704	rabbit recombinant monoclonal, 1:2000 ThermoFisher Scientific, Rockford, IL
h caspase 3	RP096-01	rabbit polyclonal, 1:200 Diagnostic Byosystems Inc., Pleasanton, CA
anti-rabbit IgG, FITC	F9887	produced in goat, affinity isolated antibody. ThermoFisher Scientific, Rockford, IL

goat anti-mouse IgG (H+L) Cross-Adsorbed Secondary Antibody, Alexa Fluor™ 488	A-11001	goat polyclonal, 1 µg/ml, ThermoFisher Scientific, Rockford, IL
goat anti-Rabbit IgG (H+L) Cross-Adsorbed Secondary Antibody, Alexa Fluor™ 647 or APC	A-21245<br>A-10931	goat polyclonal, 1 µg/ml, ThermoFisher Scientific, Rockford, IL
isotype controls	11-4752-80	ThermoFisher Scientific, Rockford, IL
goat anti-mouse IgG, HRP conjugated	12-349	goat polyclonal, 1:250, ThermoFisher Scientific, Rockford, IL

References

1. Herrera, E.; Amusquivar, E. Lipid metabolism in the fetus and the newborn. *Diabetes/metabolism research and reviews* **2000**, *16*, 202-210.
2. Gyurina, K.; Yarmak, M.; Sasi-Szabó, L.; Molnár, S.; Méhes, G.; Röszer, T. Loss of Uncoupling Protein 1 Expression in the Subcutaneous Adipose Tissue Predicts Childhood Obesity. *International Journal of Molecular Sciences* **2023**, *24*, 16706.
3. Whyte, R.K.; Bayley, H.S. Energy Metabolism of the Newborn Infant. In *Advances in nutritional research*, Draper, H.H., Ed.; Springer US: Boston, MA, 1990; pp. 79-108.
4. Persson, B. Carbohydrate and Lipid Metabolism in the Newborn Infant. *Acta Anaesthesiologica Scandinavica* **1974**, *18*.
5. Poissonnet, C.M.; Burdi, A.R.; Garn, S.M. The chronology of adipose tissue appearance and distribution in the human fetus. *Early human development* **1984**, *10*, 1-11, doi:10.1016/0378-3782(84)90106-3.
6. Ampem, G.; Röszer, T. Isolation and Characterization of Adipose Tissue Macrophages. In *Nuclear Receptors: Methods and Experimental Protocols*, Badr, M.Z., Ed.; Springer New York: New York, NY, 2019; pp. 225-236.
7. Hoang, A.C.; Yu, H.; Röszer, T. Transcriptional Landscaping Identifies a Beige Adipocyte Depot in the Newborn Mouse. *Cells* **2021**, *10*, 2368.
8. Radványi, Á.; Gyurina, K.; Rácz, E.; Kovács, I.; Méhes, G.; Röszer, T. Adipose Tissue Macrophages of the Human Fetus. *Cells* **2024**, *13*, 1787.
9. Kepple, J.D.; Liu, Y.; Kim, T.; Cero, C.; Johnson, J.W.; Rowe, G.C.; Cypess, A.M.; Habegger, K.M.; Young, M.; Hunter, C.S. The transcriptional co-regulator LDB1 is required for brown adipose function. *Molecular metabolism* **2021**, *53*, 101284, doi:10.1016/j.molmet.2021.101284.
10. Jespersen, N.Z.; Larsen, T.J.; Peijs, L.; Daugaard, S.; Homøe, P.; Loft, A.; de Jong, J.; Mathur, N.; Cannon, B.; Nedergaard, J.; et al. A classical brown adipose tissue mRNA signature partly overlaps with brite in the supraclavicular region of adult humans. *Cell Metab* **2013**, *17*, 798-805, doi:10.1016/j.cmet.2013.04.011.
11. de Jong, J.M.; Larsson, O.; Cannon, B.; Nedergaard, J. A stringent validation of mouse adipose tissue identity markers. *Am J Physiol Endocrinol Metab* **2015**, *308*, E1085-1105, doi:10.1152/ajpendo.00023.2015.
12. Rockstroh, D.; Landgraf, K.; Wagner, I.V.; Gesing, J.; Tauscher, R.; Lakowa, N.; Kiess, W.; Bühligen, U.; Wojan, M.; Till, H.; et al. Direct evidence of brown adipocytes in different fat depots in children. *PLOS ONE* **2015**, *10*, e0117841, doi:10.1371/journal.pone.0117841.
